# piR-823 and piR-20326 Differentially Regulate Stemness Programs in Ovarian Cancer Stem-like Cells

**DOI:** 10.3390/ijms27198689

**Published:** 2026-09-29

**Authors:** Ece Gumusoglu-Acar, Sude Sultan Ozdemir, Uygar Yucekaya, Ronald J. Buckanovich, Tuba Gunel

**Affiliations:** 1 Department of Molecular Biology and Genetics, Faculty of Science, Istanbul University, Bozdogankemeri St., No. 1., 34134 Istanbul, Turkey; gunel@istanbul.edu.tr; 2 Magee-Womens Research Institute, Pittsburgh, PA 15213, USA; buckanovichrj@mwri.magee.edu; 3 Department of Molecular Biotechnology and Genetics, Institute of Graduate Studies in Sciences, Istanbul University, 34116 Istanbul, Turkey; sudeozdemir85@gmail.com (S.S.O.); uygaryucekaya1@gmail.com (U.Y.); 4 Department of Internal Medicine and Department of Obstetrics, Gynecology and Reproductive Sciences, University of Pittsburgh School of Medicine, Pittsburgh, PA 15261, USA

**Keywords:** ovarian cancer, cancer stem cells, piRNA, piR-823, piR-20326

## Abstract

Ovarian cancer stem-like cells (OCSCs) are major drivers of tumor recurrence and chemoresistance. Although PIWI-interacting RNAs (piRNAs) have emerged as important regulators of cancer stem cell (CSC) biology, their roles in OCSCs remain poorly understood. We performed a systematic analysis of public datasets to identify ovarian cancer-associated piRNAs and selected piR-823 and piR-20326 for functional validation. Their expression was evaluated by quantitative reverse transcription polymerase chain reaction (qRT-PCR)following piRNA mimic and inhibitor transfections in three-dimensional (3D) ovarian cancer spheroids and ALDH^+^/CD133^+^ stem-like subpopulations isolated by fluorescence-activated cell sorting. Stemness-associated genes (*ALDH1A1*, *ALDH1A2*, *ALDH1A3*, *SOX2*, *CD133*, and *NANOG*) were analyzed, and The Cancer Genome Atlas (TCGA) transcriptomic data were used for clinical validation. Both piR-823 and piR-20326 were significantly enriched in spheroid cultures and ALDH^+^/CD133^+^ populations, supporting their association with stem-like phenotypes. Functional analyses showed that piR-823 modulation was associated with changes in multiple stemness-related markers, whereas piR-20326 showed variable effects across two-dimensional (2D) and spheroid 3D culture models. TCGA data confirmed consistent co-expression patterns among stemness-related genes in clinical samples. These findings identify piR-823 as a key regulator and piR-20326 as a modulatory component of the piRNA-mediated stemness network in ovarian cancer, supporting further investigation of the piRNA–stemness axis as a potential therapeutic target.

## 1. Introduction

Ovarian cancer (OC) is the most lethal gynecologic malignancy, with approximately 314,000 new cases and over 200,000 deaths worldwide in 2020. Although most patients initially respond to cytoreductive surgery combined with platinum- and taxane-based chemotherapy, almost 70% of patients with advanced-stage cancer relapse, and the five-year overall survival rate is under 50% [1,2].

A major contributor to the high recurrence rate is the emergence of chemoresistance, which is strongly linked to a rare subpopulation of self-renewing and tumorigenic cells known as ovarian cancer stem cells (OCSCs). OCSCs drive tumor initiation, sustain tumor progression and intratumoral heterogeneity, and contribute to therapeutic resistance through multiple mechanisms, including their capacity to remain in a quiescent state, enhanced DNA damage repair, and activation of survival signaling pathways [3,4]. In addition, interactions with the tumor microenvironment, particularly carcinoma-associated mesenchymal stem cells, have been shown to promote stem-like phenotypes and enhance chemoresistance in OC [5,6]. Targeting these stem-like populations has therefore emerged as a potential therapeutic strategy aimed at limiting tumor progression, recurrence, and treatment resistance [4,7]. OCSCs cultured as 3-dimensional (3D) spheroids in non-adherent, serum-free conditions exhibit functional enrichment and upregulated stemness markers (*SOX2*, *NANOG*, *CD133*, and *ALDH1* family) [8,9]. These factors define the stem-like phenotype and are directly linked to treatment resistance and poorer outcomes [10]. OCSCs contribute to peritoneal dissemination, a characteristic of advanced OC, in which cancer cells survive and proliferate in ascitic fluid as non-adherent spheroids, a property largely attributed to their resistance to anoikis [11].

In recent years, non-coding RNAs (ncRNAs), particularly PIWI-interacting RNAs (piRNAs), have emerged as key regulators of stemness-associated pathways and cellular processes involved in tumor progression and therapeutic resistance [12,13]. piRNAs are small RNAs (24–32 nt), originally identified in germline cells, and are associated with PIWI proteins, a subgroup of the Argonaute family [14]. Unlike microRNAs or siRNAs, piRNAs are generated by a Dicer-independent mechanism and function by silencing transposable elements, altering chromatin architecture, and regulating post-transcriptional gene expression [15].

The functional scope of the piRNA–PIWI axis has extended beyond germline maintenance, with growing evidence implicating its dysregulation in oncogenesis, metastasis, and therapy resistance across various malignancies [12]. Altered expression of PIWI proteins and piRNAs has been shown in gynecologic malignancies, such as cervical, endometrial, and OC, and is associated with poor prognosis [15]. These piRNAs modulate gene networks related to cell proliferation, apoptosis, DNA repair, and epigenetic remodeling, all relevant to cancer stem cell biology [12]. Although the role of piRNAs in cancer has been increasingly studied, their precise functions within OCSCs are unclear [16].

This study aimed to investigate the functional relevance of piRNAs to OCSC-associated phenotypes using in vitro and in silico approaches. We compared the effects of selected piRNAs on the expression of stemness-related genes in both 2D and 3D OC cell culture models. Additionally, stemness gene expression profiles from datasets representing recurrent and primary OC and OCSC-enriched groups were integrated with our in vitro findings. Our data offer new insights into the piRNA–stemness axis in OC and provide a rationale for exploring piRNAs as prospective therapeutic targets. Targeting piRNA-mediated pathways represents a promising strategy for eliminating chemoresistant cancer stem cells, thereby addressing a major obstacle in the effective treatment of OC.

## 2. Results

### 2.1. Selection of Candidate piRNAs

The identified piRNAs were ranked according to fold change (FC) values. The five most strongly downregulated and five most strongly upregulated piRNAs were selected for the next step in both down- and up-regulated piRNA lists (Table 1). These ten piRNAs were evaluated for associations with CSC biology based on their predicted target genes and studies about CSC. This integrative bioinformatic analysis identified two candidate piRNAs potentially associated with OC stemness, hsa_piRNA_30928 (piR-823) and hsa_piRNA_26089 (piR-20326), which may have functional roles in CSC biology. piR-823 is located at chromosome 19q13.32, in the C19orf18 locus, which is enriched for dbSNP polymorphisms, ENCODE cis-regulatory elements, and variations linked to OMIM diseases. piR-20326 is located on chromosome 16p13.3, where it is embedded in the VAC14 locus, which encodes a scaffold protein that is necessary for the synthesis of PI(3,5)P_2_ as well as for controlling lysosomal homeostasis and endosomal trafficking.

### 2.2. siRNA Transfection for piRNA Inhibition and Overexpression

Inhibition of piR-823 and piR-20326 (Figure 1A and Figure 1B, respectively) was most effective at 48 h of incubation following transfection. Similarly, transfection with piR-823 and piR-20326 mimics (Figure 1C and Figure 1D, respectively) exhibited optimum expression regulation at 48h post-transfection.

### 2.3. Spheroid Formation Assay

To evaluate the effect of piR-823 and piR-20326 on spheroid growth, OVSAHO cells were transiently transfected with either chemically synthesized piRNA mimics to increase intracellular piRNA levels or sequence-specific antisense inhibitors to suppress endogenous piRNA function. Following transfection, cells were transferred to ultra-low-attachment spheroid plates and cultured for 8 days with medium replacement every other day. Spheroid size and morphology were monitored daily using an inverted microscope (Figure 2A–D). Spheroid size was evaluated longitudinally within each piRNA-modulation condition. piR-20326(i)-treated cells formed spheroids throughout the culture period, whereas piR-20326(m)-treated cells formed more compact spheroids (Figure 2C,D). In contrast, spheroids in the piR-823 inhibitor condition showed an apparent increase in projected area over time, whereas those in the mimic condition displayed a more stable morphology (Figure 2A,B). For piR-823, paired analysis showed no significant change in spheroid area between Day 1 and Day 3 in the inhibitor condition (*p* = 0.262), whereas a significant increase was observed in the mimic condition (*p* = 0.019). For piR-20326, no significant change in spheroid area was observed between Day 1 and Day 3 in either the inhibitor (*p* = 0.125) or mimic (*p* = 0.996) condition (Figure 2E,F). The inhibitor and mimic conditions were analyzed independently as distinct biological interventions and were not directly compared with each other.

### 2.4. FACS

The gating strategies for the analyzed cell populations are shown in Figure S2. ALDH/CD133 double-negative (−/−) and double-positive (+/+) populations were identified and isolated by fluorescence-activated cell sorting for subsequent expression analyses. Direct counting of the collected fractions showed that approximately 7000 cells were recovered in the ALDH^+^/CD133^+^ tube. All populations were subsequently normalized to 7000 cells before RNA isolation and piRNA-expression analysis.

### 2.5. Selected piRNA Expression Profiling

The expression levels of piR-823 and piR-20326 were compared between 3D spheroid and 2D monolayer cultures (Figure 3A and Figure 3B, respectively), and between ALDH^+^/CD133^+^ and ALDH^−^/CD133^−^ OVSAHO cell populations (Figure 3C and Figure 3D, respectively). Consistent with a potential role in stemness-associated cellular states, both piR-823 (*p* = 0.038) and piR-20326 (*p* = 0.026) demonstrated significantly higher expression in 3D spheroid cultures compared with 2D monolayer controls. Similarly, both piR-823 (*p* = 0.04) and piR-20326 (*p* = 0.015) showed significantly enriched expression in the ALDH/CD133 (+/+) population relative to the ALDH/CD133 (−/−) cells.

### 2.6. Gene Expression Analysis Results in Cell Lines

The expression levels of stemness-related genes and epigenetic and EMT markers showed various profiles in different piRNA treatment conditions. The expression patterns observed under each experimental condition are described in the following sections. The distributions of the gene-expression data were additionally assessed by Q–Q plots, which are provided in Supplementary Figures S4 and S5.

#### 2.6.1. Effects of piR-823 Modulation on Stemness-Related Gene Expression

To assess the effect of piR-823 transfection on the expression of stemness-associated genes in OC cells, quantitative real-time PCR (qRT-PCR) analyses were performed in both 3D spheroid cultures (Figure 4A) and 2D adherent cultures (Figure 4B). In 3D spheroid cultures (Figure 4A), the inhibition of piR-823 significantly reduced the expression of *ALDH1A1*, *ALDH1A3*, *CD133*, and *NANOG*, while *ALDH1A2* expression was significantly increased. piR-823(m) transfection significantly elevated the expression of *ALDH1A1*, *CD133*, *NANOG*, and *SOX2*, accompanied by a significant reduction in *ALDH1A3* levels. In 2D cells (Figure 4B), the inhibition of piR-823 led to a significant decrease in *ALDH1A1*, *CD133*, *NANOG* and *SOX2* expression, while *ALDH1A2* expression was significantly elevated. piR-823 (m) transfection resulted in a significant increase in *ALDH1A1* and *NANOG* levels, along with a significant decrease in *ALDH1A2* expression. Following BH-FDR correction for multiple comparisons, the principal significant changes in stemness-associated gene expression following piR-823 regulation were retained, whereas some individual comparisons no longer remained statistically significant after adjustment. The complete raw and BH-FDR-adjusted *p*-values are provided in Supplementary Table S4.

#### 2.6.2. Effects of piR-823 Modulation on the Expression Levels of Epigenetic and EMT Markers

In 3D spheroids (Figure 5A), the inhibition of piR-823 significantly increased *DNMT3A*, *DNMT3B*, and *E-cadherin* expression, while significantly reducing Vimentin levels. In contrast, piR-823 (m) transfection significantly decreased *DNMT3A* and *DNMT3B* expression, accompanied by reduced *E-cadherin* and increased Vimentin expression. In 2D adherent cultures (Figure 5B), the inhibition of piR-823 significantly increased *DNMT3A* and *E-cadherin* expression. Conversely, piR-823 (m) transfection resulted in a significant decrease in *DNMT3A* and *E-cadherin* levels. Following BH-FDR correction, the statistical significance of the changes in epigenetic and EMT marker expression was reassessed within each culture and piRNA regulation condition. The complete raw and BH-FDR-adjusted *p*-values are provided in Supplementary Table S4.

#### 2.6.3. Effects of piR-20326 Modulation on Stemness-Related Gene Expression

To investigate the role of piR-20326 in the regulation of stemness-associated gene expression in OC cells, quantitative real-time PCR (qRT-PCR) analyses were performed in both 3D spheroid cultures and 2D adherent cultures following transfection with either a piR-20326 mimic or inhibitor (Figure 6). Following BH-FDR correction for multiple comparisons, several significant changes in stemness-associated gene expression following piR-20326 regulation remained statistically significant, whereas some individual comparisons did not retain significance after adjustment. The complete raw and BH-FDR-adjusted *p*-values are provided in Supplementary Table S5. In 3D spheroids (Figure 6A), inhibition of piR-20326 significantly reduced the expression of *ALDH1A1*, *ALDH1A3*, *NANOG*, and *SOX2*, while CD133 expression was significantly increased. Conversely, piR-20326(m) transfection significantly upregulated *ALDH1A2*, *ALDH1A3*, *NANOG*, and *SOX2* expression. In 2D adherent cultures (Figure 6B), inhibition of piR-20326 resulted in a significant increase in *ALDH1A2* expression and a significant decrease in *ALDH1A3* levels. In contrast, piR-20326 overexpression significantly increased *ALDH1A2*, *NANOG*, and *SOX2* expression.

#### 2.6.4. Effects of piR-20326 Modulation on the Expression Levels of Epigenetic and EMT Markers

In 3D spheroid cultures (Figure 7A), modulation of piR-20326 did not result in statistically significant changes in the expression of *DNMT3A*, *DNMT3B*, *E-cadherin*, or *Vimentin.* In 2D adherent cultures (Figure 7B), piR-20326(i) led to a significant decrease in *DNMT3B* expression. Conversely, piR-20326(m) reduced *DNMT3A* and *DNMT3B* levels. Following BH-FDR correction, the statistical significance of the changes in epigenetic and EMT marker expression was reassessed within each culture and piRNA regulation condition. The complete raw and BH-FDR-adjusted *p*-values are provided in Supplementary Table S5.

Figure 8 demonstrates the alterations in relative gene expression (log2 fold change) of stemness-associated markers (*SOX2*, *NANOG*, *CD133*, *ALDH1A1*, *ALDH1A2*, *ALDH1A3*), epithelial–mesenchymal transition (EMT) markers (*Vimentin*, *E-cadherin*), and epigenetic regulators (*DNMT3A*, *DNMT3B*) following modulation of piR-823 and piR-20326 in both 2D and 3D spheroid OC cell culture models. The heatmaps illustrate distinct expression patterns across the experimental conditions.

### 2.7. Overall Survival Analysis Results

Kaplan–Meier survival analyses revealed gene-specific associations with overall survival in patients with ovarian cancer (Figure 9). Higher expression of ALDH1A1 and ALDH1A2 was significantly associated with overall survival (log-rank *p* = 0.0237 and *p* = 0.0269, respectively), suggesting their potential prognostic significance. Similarly, SOX2 expression showed a statistically significant association with survival (*p* = 0.0309), supporting its potential involvement in cancer stem cell-related progression. In contrast, ALDH1A3, NANOG, and PROM1 expression levels were not significantly associated with overall survival (*p* > 0.05), indicating more limited or context-dependent prognostic associations in this cohort. To further evaluate whether the observed prognostic associations were independent of clinical features, multivariate Cox proportional hazard regression analyses were performed, adjusting for age at diagnosis, pathological stage, and tumor grade. Age was modeled as a continuous variable per 10-year increment, while pathological stage and tumor grade were included as categorical covariates. For each model, patients with missing values in any of the variables included in that analysis were excluded using a complete case approach. Adjusted hazard ratios (HRs), 95% confidence intervals (CIs), and *p*-values are presented in Figure S3.

### 2.8. Stemness Gene Expression Analysis Results in Datasets

All data from different datasets (62 ROC cases, 421 POC cases, and one OCSC sample) were evaluated according to the expression profiles of six stem cell-associated genes. The POC and ROC datasets were quantitatively analyzed using the predefined preprocessing and statistical workflow described in the Methods Section. In contrast, the OCSC dataset (GEO accession number GSM4684836) was not included in statistical comparisons or differential expression analyses with POC or ROC groups because it consisted of a single sample. The OCSC profile was used only as an independent exploratory reference to evaluate the expression pattern and correlation structure of the six selected genes. Accordingly, no statistical inference was made regarding differences between the OCSC sample and the POC or ROC groups.

In Figure 10A, the expression levels of six stem cell-related genes (*ALDH1A1*, *ALDH1A2*, *ALDH1A3*, *NANOG*, *PROM1*, and *SOX2*) were compared between cases of ROC and POC. *SOX2* (*p* = 2 × 10^−14^) and *PROM1* (*p* = 4.5 × 10^−4^) expression levels were higher in ROC compared to POC. In contrast, *ALDH1A1* expression was higher in POC compared to ROC (*p* = 0,004). No significant difference was observed between the two groups for *ALDH1A2*, *ALDH1A3*, and *NANOG*.

Figure 10B shows the Pearson correlation analysis of stem cell-related gene expression. To investigate the transcriptional similarity of stem cell-associated expression patterns among OC cohorts, correlation analyses were conducted between the OCSC, POC, and ROC datasets. The overall stem cell signature derived from OCSC cells exhibited a strong negative correlation with primary tumors (r = −0.59), whereas its correlation with recurrent tumors was nearly neutral (r = −0.04). Consistently, the POC group predominantly exhibited negative correlation values, while the ROC group showed a broader distribution of correlation values, mostly including positive correlations.

## 3. Discussion

Despite advances in OC treatment, recurrence and chemoresistance remain major clinical challenges, largely driven by cancer stem-like cell populations. Increasing evidence suggests that piRNAs play critical roles in regulating stemness and tumor plasticity; however, their functional contributions in ovarian cancer stem-like cells remain poorly defined. In this study, we aimed to identify and functionally characterize piRNAs associated with stemness-related pathways, focusing on candidates with potential regulatory roles in cancer stem cell biology. Our systematic analysis showed that piR-823 and piR-20326, both of which have previously been associated with epigenetic regulation, pluripotency, or tumor aggressiveness, were selected through subsequent filtering based on projected target genes and the existing CSC-related literature. Therefore, the study focused on these piRNAs with the highest potential to mechanistically influence CSC-related functions [18].

Spheroid formation assays are considered a functional measure of tumor-initiating ability, making them an essential tool for researching metastasis, dormancy, and chemoresistance mechanisms in the context of OC stemness [19]. In our findings, across the 8-day culture period, spheroids subjected to piR-823 inhibition showed an apparent increase in projected area over time, whereas a comparable change was not observed following piR-823 overexpression, suggesting a potential growth-promoting effect of piR-823. The piR-823 (m) may enrich the CSC subpopulation, potentially shifting the balance toward a more stem-like but less proliferative phenotype. In contrast, modulation of piR-20326 produced no appreciable change in spheroid size, indicating a limited impact on overall spheroid expansion under the tested conditions. Differences in spheroid size were interpreted as indicators of proliferative capacity and potential CSC activity but were further validated by CSC marker expression and spheroid formation assays [20,21,22]. Furthermore, piR-823 and piR-20326 were enriched in the double-positive population, supporting their association with the OCSC-like phenotype [20].

The expression patterns observed in stem-like cell-enriched conditions should be distinguished from the tissue-level expression profiles used during candidate selection. Although piR-823 and piR-20326 showed lower expression in the ovarian cancer dataset relative to ovarian tissue, both piRNAs were relatively enriched in 3D spheroids and in the ALDH+/CD133+ population in OVSAHO cells. These observations are not necessarily contradictory because bulk tumor expression represents the average signal from multiple cellular populations, whereas 3D spheroid culture and ALDH/CD133-based enrichment specifically examine cell populations with stem-like characteristics. A transcript that is enriched within a relatively small cellular subpopulation may therefore remain reduced at the level of the bulk tumor. In the present study, the findings presented in Figure 3 are consequently interpreted as enrichment of piR-823 and piR-20326 within stemness-associated cellular conditions rather than as evidence of generalized upregulation in ovarian cancer.

PiR-823 is an extensively studied piRNA in oncology, exhibiting the capacity to influence stemness, cellular viability, and chemoresistance in breast, colorectal, and hematologic cancers [23]. piR-823 has been reported to enhance its oncogenic effects by modulating factors such as DNMTs, HSP27, and VEGF, thereby influencing both epigenetic programming and interactions within the tumor microenvironment [9]. Our findings showed that piR-823 regulation was associated with changes in several stemness-related markers, including *ALDH1A1*, *CD133*, and *NANOG*. However, the magnitude and direction of these changes varied among individual markers and between 2D monolayer and 3D spheroid cultures. These findings indicate that the effects associated with piR-823 regulation depend on the cellular culture condition rather than representing a uniform stemness-related response. Previous studies have shown that piR-823 promotes cancer stem cell phenotypes by enhancing pluripotency factors and increasing ALDH-positive populations [24]. Consistent with these findings, our results extend the stemness-promoting role of piR-823 to OC.

In addition to its role in stemness, piR-823 has been implicated in epigenetic regulation through *DNMT3A*/*DNMT3B*-mediated DNA methylation [25,26]. This epigenetic role is further supported by previous studies showing that piR-823 promotes tumorigenesis through DNMT-mediated silencing of tumor suppressor genes, consistent with its localization in the regulatory element-rich 19q13.32 region [24,25,26,27]. Consistent with this mechanism, piR-823 inhibition increased *DNMT3A* expression while reducing *VIMENTIN* and increasing *E-CADHERIN* levels, whereas piR-823 overexpression was associated with EMT-related changes. Together with previous reports linking piR-823, *PIWIL1*, and *DNMT3B* to stemness and CDH1 repression in OC [27], our findings support a role for piR-823 in coordinating epigenetic remodeling, EMT, and stemness-associated cellular plasticity.

Similarly, although less extensively characterized, piR-20326 has been linked to oncogenic signaling and is predicted to regulate genes involved in cellular plasticity and therapeutic response [9]. Its localization within the VAC14 locus at 16p13.3 further suggests a connection to vesicular trafficking and lysosomal pathways, which are increasingly recognized as important regulators of cancer cell adaptability and stemness [28,29]. Together with TCGA-based transcriptional evidence, these findings support the functional integration of OC-associated piRNAs into regulatory networks.

piR-20326 also showed variable effects on stemness-associated genes. Although *SOX2* and *NANOG* were responsive to piR-20326 modulation in several conditions, other markers, including *CD133* and *ALDH1A3*, showed different responses depending on the experimental condition. These findings indicate that the effects of piR-20326, similarly to those of piR-823, vary according to the marker examined and the culture condition. While *SOX2* and *NANOG* show consistent changes under overexpression and inhibition conditions, other markers such as *CD133* and *ALDH1A3* display variable responses, indicating a more specific regulatory role. Moreover, the absence of significant changes in DNMT expression suggests that piR-20326 may have a more limited impact on epigenetic remodeling compared to piR-823. Collectively, these findings suggest that piR-20326 functions as a modulatory component of the piRNA regulatory network, contributing to cellular plasticity and adaptive responses in OC stem-like cells.

Although the functions of piRNAs involve their association with PIWI-family proteins, direct physical binding of piR-823 or piR-20326 to a specific PIWI protein was not examined in OVSAHO cells in the present study. Previous cancer studies have demonstrated functional piRNA–PIWI associations for several piRNAs, supporting the biological relevance of the PIWI–piRNA pathway in cancer. For piR-823, previous studies have additionally demonstrated effects on cancer stemness, DNMT expression, DNA methylation, and Wnt signaling, and a direct RNA–protein association between piR-823 and HSF1 has been demonstrated in luminal breast cancer [6]. Recent ovarian cancer data have also identified an association between piR-823 and *PIWIL1* expression and proposed a potential relationship between these molecules. However, direct biochemical evidence establishing a piR-823–PIWI complex in ovarian cancer cells, or a piR-20326–PIWI complex, remains unavailable. Direct analysis of piRNA–PIWI complexes by approaches such as RNA immunoprecipitation followed by piRNA-specific detection will be required to establish the PIWI dependence of these effects.

OCSCs have been associated with resistance to platinum- and taxane-based chemotherapy through several properties, including quiescence, enhanced DNA damage repair, drug efflux, and activation of survival pathways. In the present study, piR-823 and piR-20326 were enriched under stemness-associated conditions, and their regulation was associated with changes in several markers related to the OCSC phenotype. These observations provide a basis for examining whether these piRNAs also influence treatment response. Direct assessment of chemotherapy sensitivity was beyond the scope of the present experiments. Future studies should therefore evaluate cell viability and apoptosis following piRNA modulation in combination with cisplatin or paclitaxel.

This study has several limitations. All functional experiments were performed in vitro using established OC cell lines and 3D spheroid models, which may not fully recapitulate in vivo tumor complexity. The changes observed following piR-823 and piR-20326 inhibition or overexpression in the present study are interpreted as effects associated with piRNA regulation and not as evidence of a confirmed PIWI-dependent mechanism. A further limitation concerns the purity of the sorted cell populations. Although ALDH^+^/CD133^+^ cells were sorted and normalized to equal cell numbers before RNA isolation, post-sort flow-cytometric re-analysis was not performed. In addition, the lack of patient-derived samples limits the clinical relevance of our findings and prevents direct correlation with clinical outcomes such as survival or treatment response. Future studies incorporating in vivo models, patient-derived systems, and genome-wide target analyses will be essential to further elucidate the functional roles and therapeutic potential of piRNAs in OC.

## 4. Materials and Methods

### 4.1. PiRNA Selection

#### 4.1.1. piRNA Sequencing Data Analysis

To identify piRNA expression differences in OC, two datasets were obtained from the piRNAQuest v.2 database: one containing piRNA expression profiles across different cancer types (http://dibresources.jcbose.ac.in/zhumur/pirnaquest2/download_original.php (accessed on 10 January 2026)), and another containing tissue-specific piRNA expression profiles derived from normal human tissues (http://dibresources.jcbose.ac.in/zhumur/pirnaquest2/download_original.php (accessed on 10 January 2026)). Both datasets were imported into R (v4.4.1) for further analysis. From these, piRNAs listed under “Ovarian Cancer” in the cancer-associated dataset and those listed under “Ovary” in the tissue-specific dataset were selected. The two filtered datasets were then compared to identify common piRNAs whose expression levels in cancer and normal tissue were evaluated, and fold change values were calculated to quantify differential expression.

#### 4.1.2. CSC-Related piRNA Selection

CSC-related piRNAs were selected from the common piRNAs identified in both the OC and ovarian tissue datasets. First, the gene targets of these piRNAs were retrieved from the piRNAQuest v.2 database and matched with the previously identified common piRNAs. Then, Gene Ontology (GO) analysis was performed to examine the biological processes associated with these genes. The analysis focused on biological terms related to CSC, including ‘cancer stem cell,’ ‘CSC,’ ‘stemness,’ ‘self-renewal,’ ‘tumor-initiating,’ ‘sphere formation,’ ‘tumorigenic potential,’ ‘stem cell-like,’ ‘stem cell phenotype,’ ‘cancer stemness,’ ‘tumor stem,’ ‘recurrence,’ ‘quiescence,’ ‘organoid,’ ‘spheroid,’ and ‘chemoresistance.’ piRNAs linked to genes involved in these processes were selected as CSC-related piRNAs. These terms were also used as keywords to identify relevant studies on the relationship between piRNAs and cancer stem cells. piRNAs that were directly reported to be associated with CSC biology in published research were also identified. After all these analyses, two piRNAs, hsa_piRNA_30928 (piR-823) and hsa_piRNA_26089 (piR-20326), were selected for further steps. For genomic contextualization of candidate piRNAs, the UCSC Genome Browser (hg38 assembly) was utilized to determine chromosomal localization and to inspect surrounding regulatory features. Genomic tracks corresponding to histone modifications (H3K4me3 and H3K27ac) were integrated to annotate promoter and enhancer activity at piRNA loci.

### 4.2. Cell Culture

OVSAHO cells (Cellosaurus ID: CVCL_3114) were cultured in complete RPMI (10% Fetal Bovine Serum (Gibco, Thermo Fisher Scientific, Waltham, MA, USA), 1% Penicillin/Streptomycin (ibco, Thermo Fisher Scientific, Waltham, MA, USA)) at 37 °C in 5% CO_2_ until the cells were 80% confluent. The cells were collected using 0.25% trypsin (phenol red) (Gibco, ThermoFisher Scientific, USA) and were kept in RLT Lysis Buffer (QIAGEN, Hilden, Germany). All lysates were stored at −80 °C until RNA isolation.

### 4.3. siRNA-Mediated Transfection

The oligonucleotides contained 2′-O-methyl modifications used to inhibit (i) or mimic (m) the target piRNAs [30]. Endogenous piRNA activity was suppressed using sequence-specific antisense oligonucleotide (ASO) inhibitors targeting piR-823 and piR-20326, while synthetic piRNA mimics were used to increase intracellular piRNA levels. A final concentration of 50 nM was used in all in vitro assays [31]. All siRNA sequences are given in Table S1. Transfections were performed in serum-free conditions using Xfect RNA Transfection Reagent (Takara Bio USA, Inc., San Jose, CA, USA) following the manufacturer’s instructions. For each experimental condition, cells derived from a single cell-culture preparation were distributed into two parallel wells and subjected to the same transfection procedure. Cells were incubated for 24, 48, and 72 h post-transfection. Total RNA was collected at each time point, and the expression levels of piR-823 and piR-20326 were measured by qRT-PCR, with U6 used as the endogenous control.

### 4.4. 3D Spheroid Formation Assay

A six-well ‘ultra-low attachment flask’ (Corning Incorporated, Corning, NY, USA) and serum-free medium were used to generate spheroids from plated cells (1 × 10^6^ cells/well). The spheroid medium contained 1X B27, 20 ng/mL EGF, 10 ng/mL bFGF, 1 ng/mL hydrocortisone, 5 µg/mL insulin, 100 µM β-mercaptoethanol, 20 µg/mL gentamycin, and 1X Penicillin/Streptomycin in 500 mL ‘Mammary Epithelium Basal Medium’ (MEBM) (without phenol red) (Lonza, Basel, Switzerland). Spheroids were cultured at 37 °C in 5% CO_2_ for up to 8 days and fed with 500 µL spheroid medium every two days. Harvested spheres were digested in RLT Buffer (QIAGEN) and stored at −80 °C until RNA isolation.

For the effects of piR-823 and piR-20326 on spheroid formation, 7.5 × 10^3^ cells were plated on 96-well cell culture plates, and after 24 h of culturing, inhibitor and mimic siRNAs of both piRNAs were transfected into cells according to optimised transfection conditions. The mock-transfected cells (only transfection buffer) and control cells were also plated with the same number of cells. After 48 h of incubation, cells from all experimental groups were transferred to “AkuraTM 384 Spheroid microplate” (InSphero AG, Schlieren, Switzerland) in spheroid medium. For each experimental condition, spheroid assays were performed in two parallel culture wells prepared from the same experimental cell preparation. Spheroids were imaged every 24 h for 8 days. To measure the spheroid size in each image, spheroid analysis was performed using image processing. After images were converted to grayscale and analyzed in R, the spheroids were segmented using the Otsu thresholding method. “Bwlabel” was used to label and count individual spheroids, while “computeFeatures.shape” was used to calculate morphological parameters, including area. A predefined scale bar was used to convert pixel area to µm^2^, from which radius and diameter were calculated. Spheroid growth was assessed by comparing the projected area between days 1 and 3 within each piRNA modulation condition (inhibitory and mimic) for piR-823 and piR-20326. Since spheroid measurements on Days 1 and 3 were obtained from the same individual spheroids, paired Student’s *t*-tests were used to assess changes in spheroid area over time within each piRNA modulation condition. The inhibitor and mimic conditions were analyzed independently as distinct biological interventions and were not directly compared with each other.

### 4.5. Fluorescence-Activated Cell Sorting (FACS)

ALDH/CD133 (+/+), (+/−) and (−/−) cells were labeled and sorted according to the manufacturer’s instructions for the ALDEFLUOR Kit “Aldefluor Kit for ALDH Assays” (StemCell Technologies, Vancouver, BC, Canada). An APC-conjugated anti-CD133 antibody (Miltenyi Biotec, Bergisch Gladbach, Germany) was used at a ratio of 1/250. Four controls were prepared, “unstained”, “DAPI-stained”, “CD133+ antibody isotype”, and “ALDH inhibitor-DEAB”, according to the instructions of the kit. Four-way cell sorting was performed with the BD FACS Aria Fusion device. The data were analyzed using FlowJo software v10.6.2. After sorting, cells were washed with DPBS and counted using a Moxi Cell Counter (Orflo Technologies, Seattle, WA, USA). All cell populations were normalized to the cell number of the fraction with the lowest post-sort recovery, lysed with RLT buffer (QIAGEN, Hilden, Germany), and mixed by pipetting until the entire pellet was homogeneously resuspended. The entire cell suspension was stored at −80 °C until RNA isolation in 350 µL of RLT Buffer (QIAGEN, Hilden, Germany).

### 4.6. RNA Extraction and piRNA Expression Analysis

Total RNA was isolated using the RNeasy Mini Kit (QIAGEN, Germany), with the ethanol modification recommended by the manufacturer for improved recovery of small RNA species. RNA was eluted with 30 µL of ‘RNase-free water,’ and NanoDrop One (Thermo Fisher Scientific, Waltham, MA, USA) was used for RNA concentration determination. RNA samples were stored at −80 °C until qPCR was performed. For each experimental condition, cells derived from a single cell-culture preparation were distributed into two parallel wells and subjected to the same transfection procedure. The wells were processed separately for RNA isolation and cDNA synthesis, and each cDNA sample was analyzed in three technical qPCR replicates.

piR-823 and piR-20326 were quantified using a stem–loop RT-qPCR method (Figure S1) [17]. The stem–loop RT primers were hybridised to a piRNA molecule and then reverse-transcribed with a reverse transcriptase. Next, the RT products were quantified using conventional SYBR Green qPCR. For data normalization, U6 small RNA was selected as a control RNA. All primer sequences are listed in Table S2.

During cDNA synthesis, RNAs were diluted to a concentration of 50ng/ul. The stem–loop primers were used for the “PrimeScript™ Reagent Kit Synthesis System for RT-PCR (Takara Bio USA, Inc., San Jose, CA, USA)” piRNA analysis, and a U6-specific reverse primer was used for the endogenous control. “SsoAdvanced Universal SYBR Green Supermix (Bio-Rad Laboratories, Hercules, CA, USA)” master mix was used in the qRT-PCR step, completed using 2.5 µM forward and reverse primers. “CFX96 Real Time System (BIO-RAD, USA)” was used for qPCR. The expression differences of piRNAs compared to U6 were calculated using the 2^−∆∆Ct^ method. Cells transfected with a non-targeting scrambled RNA sequence served as controls. Comparative statistical analyses between groups were performed utilizing Student’s *t*-test.

### 4.7. CSC-Related Gene Expression Analysis

Diluted RNAs (50ng/ul) and random primers were used according to the “PrimeScript™ RT Master Mix Synthesis System for RT-PCR (Takara Bio USA, Inc., San Jose, CA, USA)” (Table S3). “SsoAdvanced Universal SYBR Green Supermix (Bio-Rad Laboratories, Hercules, CA, USA)” master mix was used for mRNA expression analysis of genes. According to the kit’s instructions, the qPCR step was completed using 2.5 µM forward and reverse primers. “CFX96 Real-Time System (Bio-Rad Laboratories, Hercules, CA, USA)” was used for qPCR. The expression differences in cancer stem cell-specific genes compared to control genes were calculated using the 2^−∆∆Ct^ method. Cells transfected with a non-targeting scrambled RNA sequence served as controls. Normality of the gene-expression data was evaluated in GraphPad Prism (Version 10.3.1) using the Shapiro–Wilk test and graphical assessment with normal Q–Q plots. Given the small sample size, the D’Agostino–Pearson, Anderson–Darling, and Kolmogorov–Smirnov tests could not be calculated by GraphPad Prism for these datasets. Normal Q–Q plots were therefore additionally examined to visually assess the distribution of the observations relative to the expected normal distribution. Q–Q plots were generated separately for each analyzed gene and culture condition (3D spheroid and 2D monolayer), including the corresponding inhibitor-transfected, control, and mimic-transfected samples. Comparative statistical analyses between groups were performed utilizing Student’s *t*-test. The 2D monolayer and 3D spheroid cultures were analyzed as distinct experimental models. Within each culture condition, piRNA inhibition and overexpression were evaluated independently relative to their corresponding controls; therefore, inhibitor-versus-mimic and 2D-versus-3D comparisons were not defined as primary hypothesis tests for these gene-expression analyses. To account for multiple comparisons across the analyzed genes, raw *p*-values were adjusted using the Benjamini–Hochberg false discovery rate (BH-FDR) procedure. Hypothesis families were defined separately according to the piRNA analyzed (piR-823 or piR-20326), culture condition (2D monolayer or 3D spheroid), regulation condition (inhibitor versus its corresponding control or mimic versus its corresponding control), and predefined gene panel. The stemness-related gene panel comprised six tests (*ALDH1A1*, *ALDH1A2*, *ALDH1A3*, *SOX2*, *NANOG*, and *CD133/PROM1*), whereas the epigenetic/EMT panel comprised four tests (*DNMT3A*, *DNMT3B*, *E-cadherin/CDH1*, and *Vimentin/VIM*). Accordingly, BH-FDR correction was applied independently to each family of six or four tests, respectively. Inhibitor-versus-mimic and 2D-versus-3D comparisons were not included in these families because they were not defined as primary hypothesis tests for the gene-expression analyses. An FDR-adjusted *p*-value < 0.05 was considered statistically significant.

### 4.8. Overall Survival Analysis

To determine the potential prognostic significance of stem cell-related genes in ovarian cancer, overall survival (OS) analyses were performed using publicly available datasets from The Cancer Genome Atlas (TCGA), specifically the TCGA-OV cohort (Ovarian Serous Cystadenocarcinoma; project ID: TCGA-OV). Gene expression data and related clinical survival information were obtained from the Genomic Data Commons (GDC) Data Portal. Patients were divided into high- and low-expression cohorts based on the median expression value for each gene. Kaplan–Meier survival curves were constructed to compare OS between groups, and statistical significance was assessed using the log-rank test. A *p*-value less than 0.05 was considered statistically significant. Survival studies were performed for *ALDH1A1*, *ALDH1A2*, *ALDH1A3*, *NANOG*, *SOX2*, and *PROM1* (*CD133*). To more thoroughly evaluate the independent prognostic association of each stem cell-associated gene, multivariate Cox proportional hazards regression analysis was performed, adjusted for available clinical covariates, and adjusted hazard ratios (HRs) were calculated with 95% confidence intervals and corresponding *p*-values. All statistical analyses were performed using Python version 3.13. Statistical analyses and data processing were conducted using the relevant Python packages.

### 4.9. Expression Profiling of Stemness-Related Genes Across Public Datasets

Expression profiles of six stem cell-associated genes, *ALDH1A1*, *ALDH1A2*, *ALDH1A3*, *SOX2*, *NANOG*, and *PROM1*, were examined across primary ovarian cancer (POC), recurrent ovarian cancer (ROC), and ovarian cancer stem cell (OCSC) datasets. The analysis included data collection, preprocessing, normalization, statistical comparison, correlation analysis, and visualization.

RNA sequencing gene expression data from 421 POC samples were obtained from The Cancer Genome Atlas (TCGA), while expression data from 62 ROC samples were obtained from publicly available transcriptomic datasets. The POC and ROC datasets were comparatively analyzed to investigate differences in expression of the six selected stem cell-associated genes between primary and recurrent ovarian cancer.

For the comparative analysis of the POC and ROC cohorts, expression data were processed using a consistent workflow to enhance comparability between the datasets. Genes with very low expression levels (FPKM < 10 in more than 80% of samples) were excluded before further analysis. The remaining expression values were subjected to log_2_ transformation using log_2_(FPKM + 1) to reduce the effect of highly expressed genes and balance the variance. The transformed expression values were then used for statistical analysis and visualization. Differential expression between POC and ROC groups was assessed using statistical tests selected according to the distribution characteristics of the data and unequal sample sizes between groups. Multiple test correction was performed using the false discovery rate (FDR) approach. Genes were considered significantly differentially expressed when the adjusted *p*-value was < 0.05 and the absolute log_2_ fold change was > 1. Additional pairwise correlation analyses were performed among the six stem cell-associated genes to investigate potential coordinated expression patterns.

An independent OCSC expression profile was obtained from the Gene Expression Omnibus (GEO) under accession number GSM4684836. Since this dataset represents a single OCSC sample, it was not included in the statistical comparison or differential expression analysis between POC and ROC cohorts. Specifically, pooled statistical analyses or direct group-based differential expression tests were not performed by combining the single OCSC sample with POC and ROC cohorts. Instead, the OCSC expression profile was used as a standalone exploratory dataset to examine the expression patterns and correlation structure of six stem cell-associated genes selected within the OCSC context.

Because the OCSC dataset consisted of a single sample and was used solely for exploratory purposes, the analysis focused on gene expression patterns and correlations rather than direct quantitative comparisons across cohorts. The three datasets were therefore analyzed separately and were not combined into a single expression matrix, and no cross-cohort batch-effect correction was applied. Accordingly, interpretations including the OCSC dataset were limited to descriptive and exploratory analyses, and no statistical inferences were made regarding differential expression between the OCSC sample and POC or ROC groups.

## 5. Conclusions

In conclusion, our findings identify piR-823 and piR-20326 as candidate piRNAs associated with stemness-related pathways in ovarian cancer stem-like cells. Our results show that piR-823 modulation is associated with changes in markers of remodeling, EMT, and cancer stemness, with responses varying according to the marker examined and the culture condition. Its enrichment in spheroid cultures and ALDH^+^/CD133^+^ populations, together with the changes observed in stemness-associated genes such as *NANOG* and ALDH family members, supports its involvement in stemness-associated cellular states. These findings suggest that piR-823 may contribute to cellular plasticity and features associated with therapy resistance in ovarian cancer. piR-20326 showed more variable effects across stemness-associated markers and culture conditions, suggesting a distinct role in ovarian cancer stem-like cells. Taken together, these findings support further investigation of piR-823 and piR-20326 as components of stemness-associated regulation in ovarian cancer and as potential molecular targets in approaches directed against ovarian cancer stem-like cells.

## Figures and Tables

**Figure 1:**
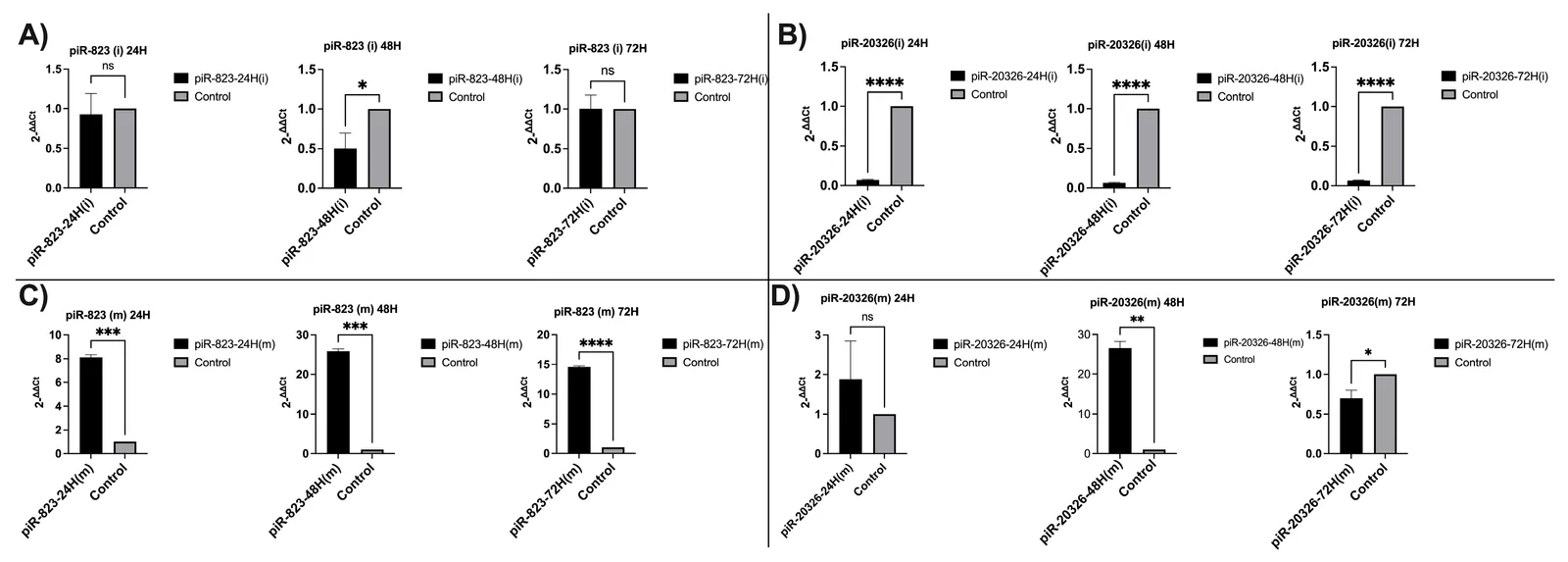
(**A**,**B**) Optimization of incubation time for piRNA inhibition. qRT-PCR analysis of piR-823 (**A**) and piR-20326 (**B**) expression levels in control cells and cells transfected with sequence-specific piRNA inhibitors at different incubation times (24 h, 48 h, and 72 h). (**C**,**D**) Optimization of incubation time for piRNA overexpression. qRT-PCR analysis of piR-823 (**C**) and piR-20326 (**D**) expression levels in control cells and cells transfected with synthetic piRNA mimics at the indicated incubation times. (* *p* < 0.05, ** *p* < 0.01, *** *p* < 0.001, **** *p* < 0.0001, ns: non-significant).

**Figure 2:**
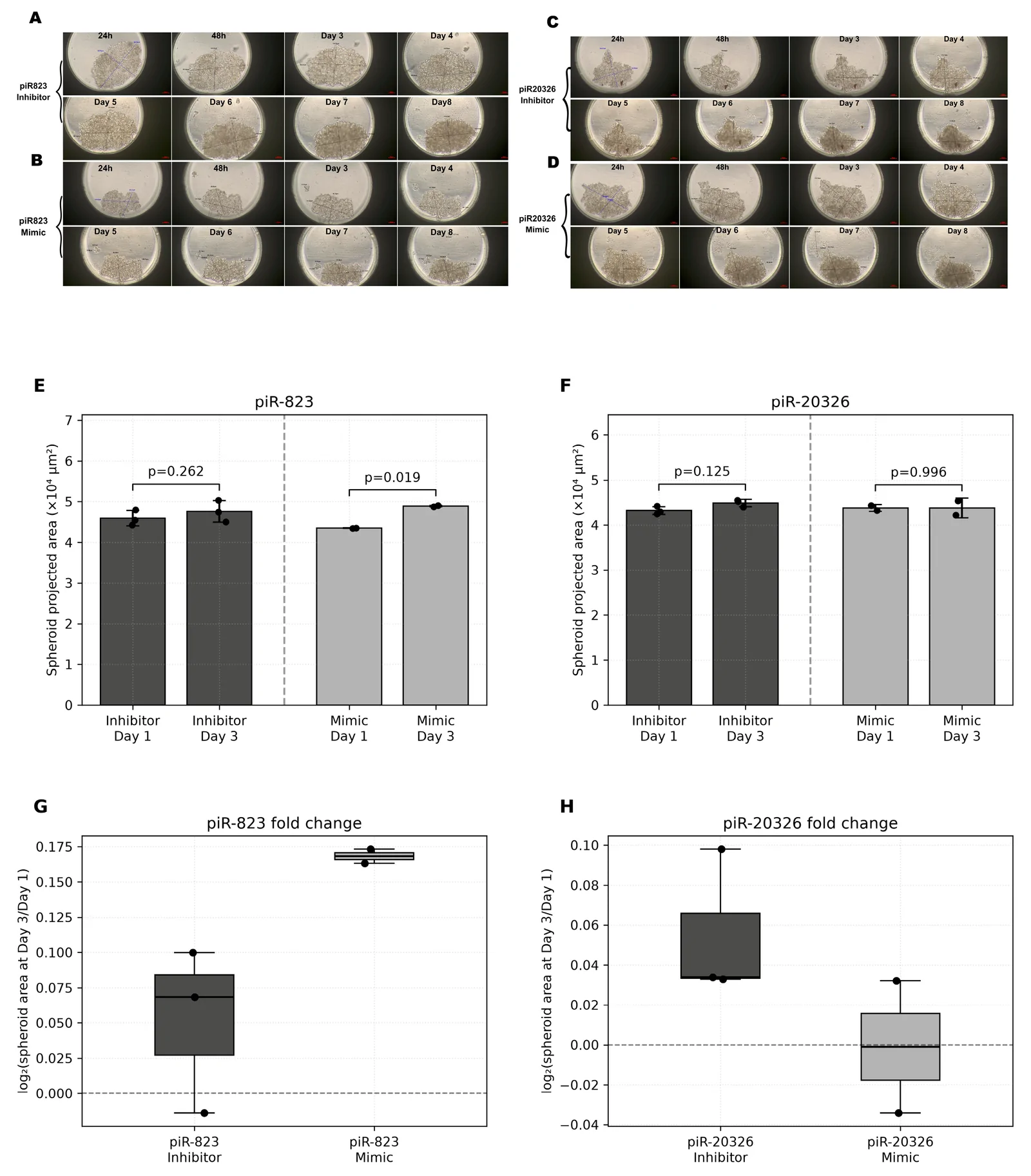
Spheroid growth following piR-823 and piR-20326 modulation in ovarian cancer cells. (**A**,**B**) Representative phase-contrast images of spheroids following transfection with the piR-823 inhibitor (**A**) or mimic (**B**) over an 8-day culture period. (**C**,**D**) Representative phase-contrast images of spheroids following transfection with the piR-20326 inhibitor (**C**) or mimic (**D**) over an 8-day culture period. (**E**) Quantification of spheroid projected area (×10^4^ µm^2^) in the piR-823 inhibitor and mimic conditions at Day 1 and Day 3. Each dot represents an individual spheroid measurement at the indicated time point. Changes in spheroid area over time were evaluated independently within each modulation condition using paired measurements from the same spheroids. No significant change was observed in the inhibitor condition (*p* = 0.262), whereas a significant increase was observed in the mimic condition (*p* = 0.019). (**F**) Quantification of spheroid projected area (×10^4^ µm^2^) in the piR-20326 inhibitor and mimic conditions at Day 1 and Day 3. Each dot represents an individual spheroid measurement at the indicated time point. Paired analysis revealed no significant change in spheroid area over time in either the inhibitor (*p* = 0.125) or mimic (*p* = 0.996) condition. Data in panels (**E**,**F**) are presented as mean ± SD. (**G**,**H**) Relative spheroid growth, expressed as log_2_ fold change in spheroid projected area between Day 3 and Day 1 [log_2_(Day 3/Day 1)], for piR-823 (**G**) and piR-20326 (**H**) modulation conditions. Each dot represents the log_2_ fold change calculated from paired Day 1 and Day 3 measurements of the same individual spheroid. Box plots depict the distribution of individual spheroid growth rates within the inhibitor and mimic conditions. These conditions represent distinct biological interventions and were evaluated independently rather than as direct mutual controls; therefore, no direct statistical comparison was performed between inhibitor- and mimic-treated spheroids. The dashed horizontal line at y = 0 indicates no change in spheroid area between Day 1 and Day 3 (log2 fold change = 0); values above the line indicate growth, while values below indicate a decrease in spheroid area.

**Figure 3:**
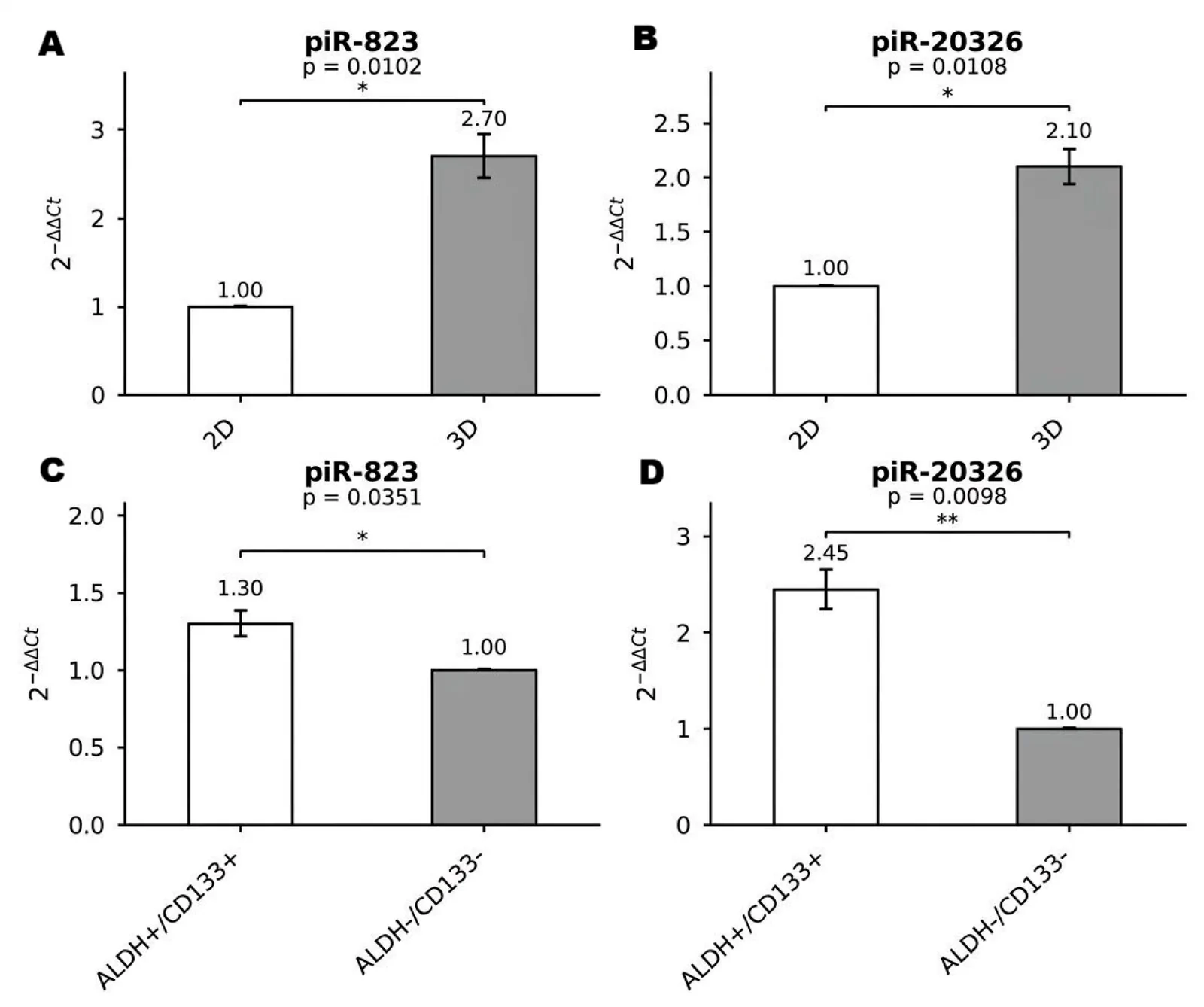
The expression of piRNAs in 3D spheroid versus 2D monolayer cultures and in ALDH^+^/CD133^+^ versus ALDH^−^/CD133^−^ OVSAHO cell populations. (**A**) piR-823 expression in 3D, (**B**) piR-20326 expression in 3D, (**C**) piR-823 expression in ALDH/CD133 (+/+), (**D**) piR-20326 expression in ALDH/CD133 (+/+). (* *p* < 0.05, ** *p* < 0.01).

**Figure 4:**
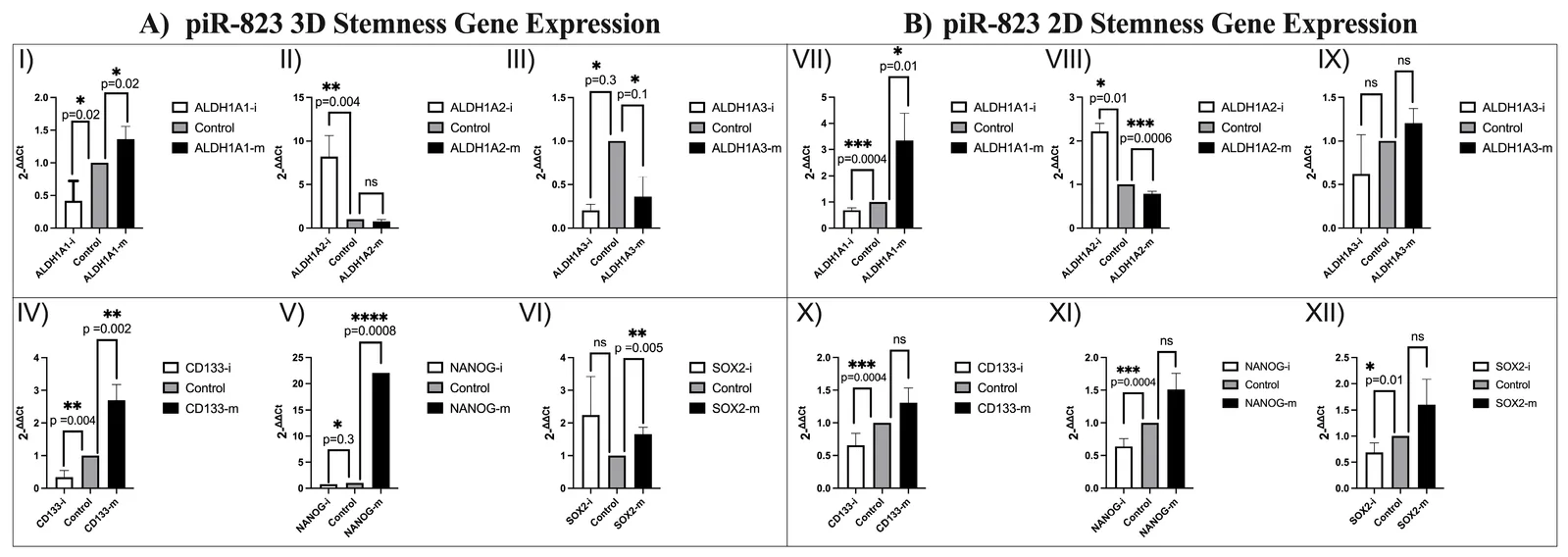
The effect of piR-823 modulation on the expression of stemness-related genes in ovarian cancer cells cultured in 3D spheroid (**A**) and 2D monolayer (**B**) conditions. (**I**–**VI**) piR-823 expression modulation in 3D spheroid cultures: (**I**) *ALDH1A1*, (**II**) *ALDH1A2*, (**III**) *ALDH1A3*, (**IV**) *CD133 (PROM1)*, (**V**) *NANOG*, and (**VI**) *SOX2*. (**VII**–**XII**) piR-823 expression modulation in 2D cultures: (**VII**) *ALDH1A1*, (**VIII**) *ALDH1A2*, (**IX**) *ALDH1A3*, (**X**) *CD133 (PROM1)*, (**XI**) *NANOG*, and (**XII**) *SOX2*. (* *p* < 0.05, ** *p* < 0.01, *** *p* < 0.001, **** *p* < 0.000, ns: not significant).

**Figure 5:**
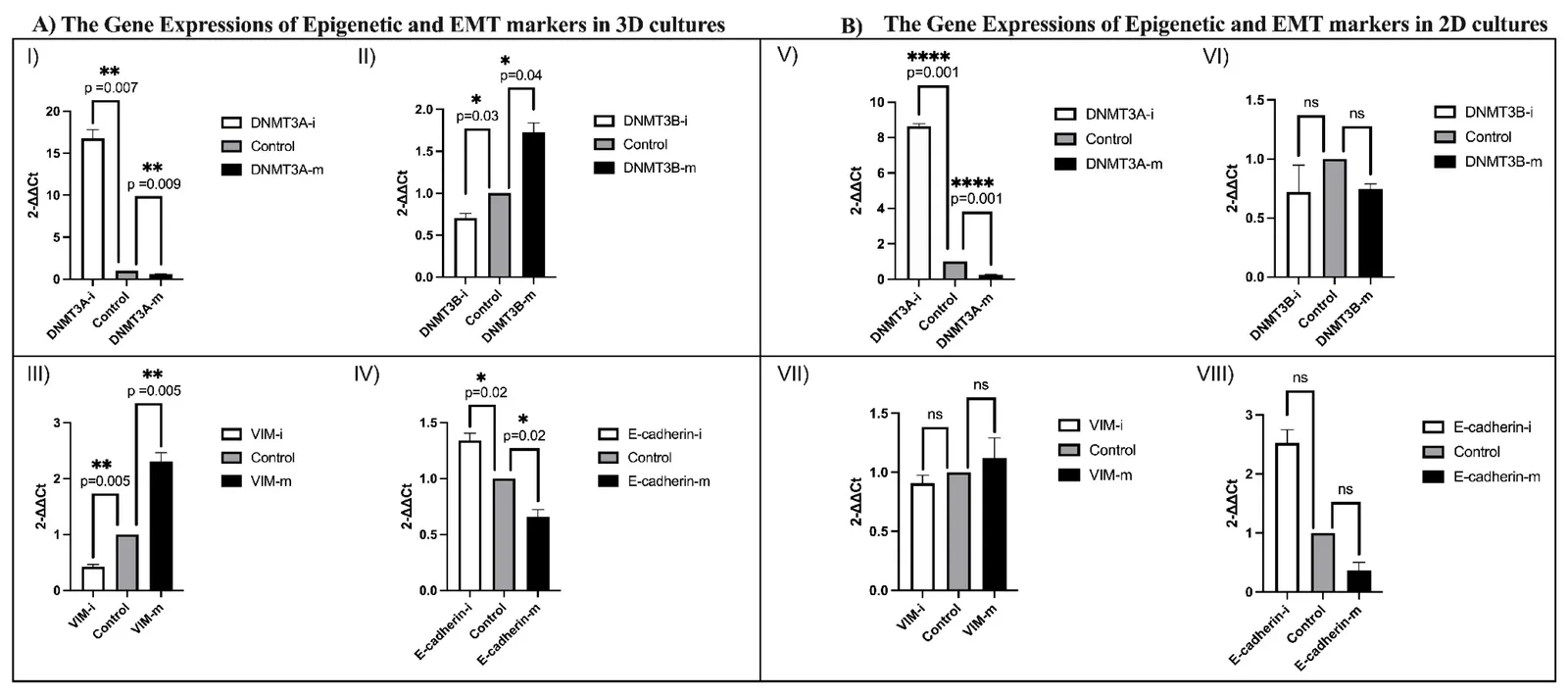
The effect of piR-823 modulation on the expression of epigenetic and EMT markers in ovarian cancer cells cultured in 3D spheroid (**A**) and 2D monolayer (**B**) conditions (**I**–**IV**) Gene expression analysis in 3D cultures: (**I**) *DNMT3A*, (**II**) *DNMT3B*, (**III**) *VIM*, and (**IV**) *E-cadherin*. (**V**–**VIII**) Gene expression analysis in 2D cultures: (**V**) *DNMT3A*, (**VI**) *DNMT3B*, (**VII**) *VIM*, and (**VIII**) *E-cadherin*. (* *p* < 0.05, ** *p* < 0.01, **** *p* < 0.0001, ns: not significant).

**Figure 6:**
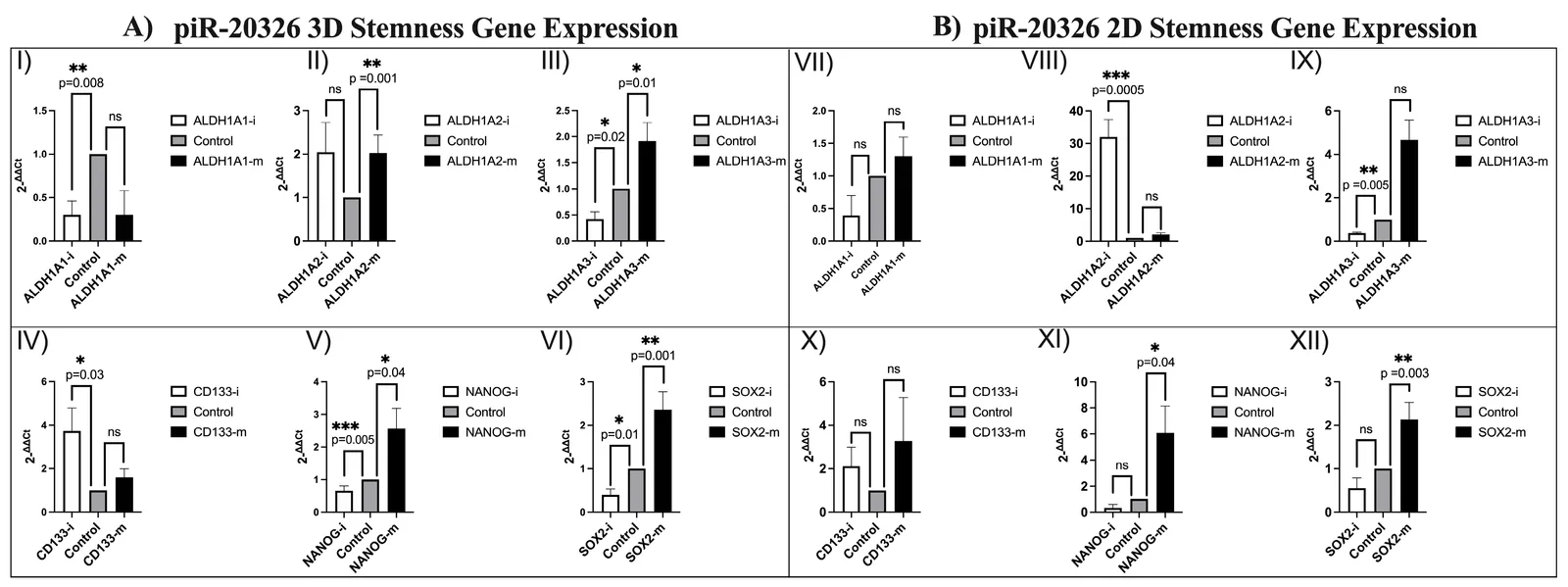
The effect of piR-20326 modulation on the expression of stemness-related genes in ovarian cancer cells cultured in 3D spheroid (**A**) and 2D monolayer (**B**) conditions. (**I**–**VI**) piR-823 expression modulation in 3D spheroid cultures: (**I**) *ALDH1A1*, (**II**) *ALDH1A2*, (**III**) *ALDH1A3*, (**IV**) *CD133 (PROM1)*, (**V**) *NANOG*, and (**VI**) *SOX2*. (**VII**–**XII**) piR-823 expression modulation in 2D cultures: (**VII**) *ALDH1A1*, (**VIII**) *ALDH1A2*, (**IX**) *ALDH1A3*, (**X**) *CD133 (PROM1)*, (**XI**) *NANOG*, and (**XII**) *SOX2*. (* *p* < 0.05, ** *p* < 0.01, *** *p* < 0.001, ns: not significant).

**Figure 7:**
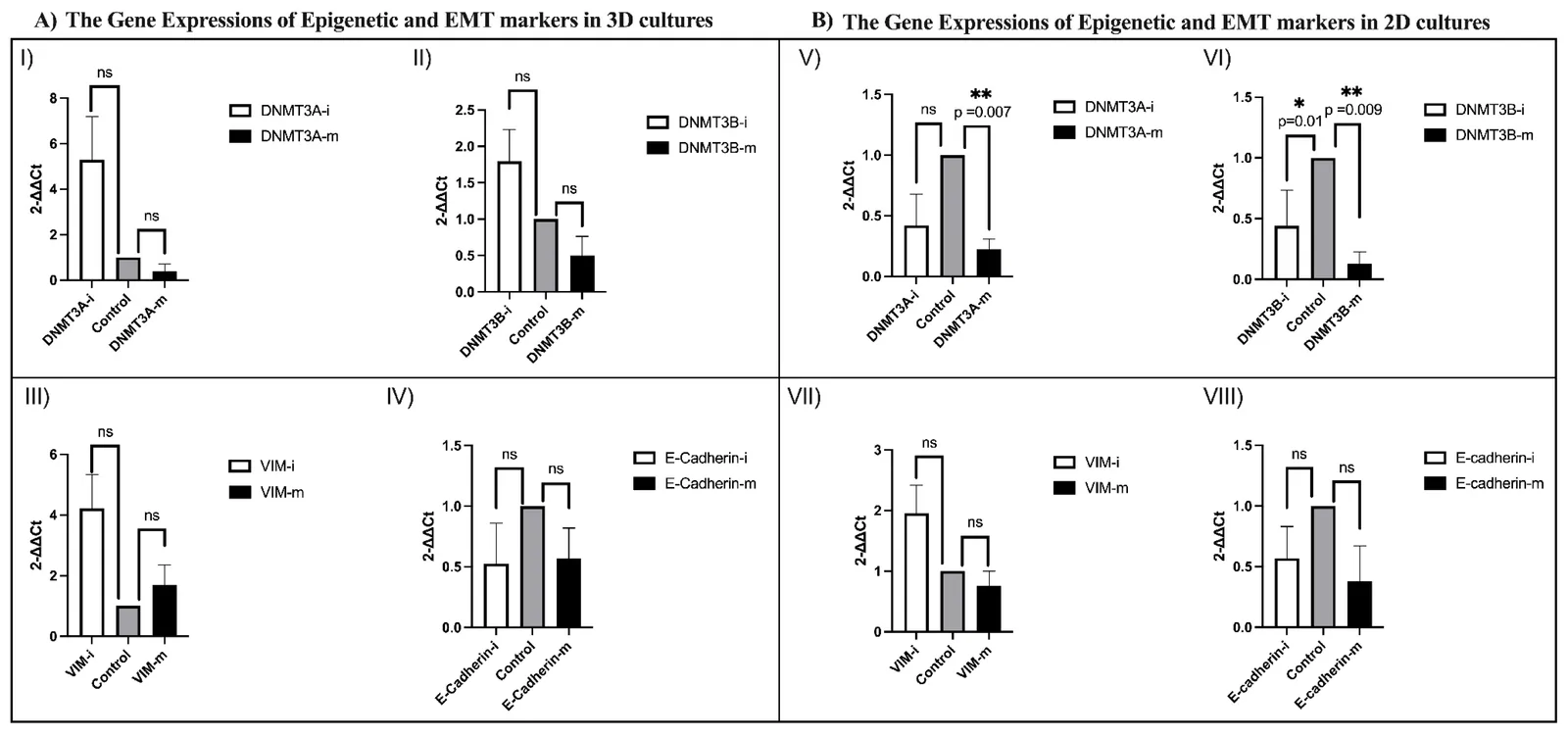
The effect of piR-20326 modulation on the expression of epigenetic and EMT markers in ovarian cancer cells cultured in 3D spheroid (**A**) and 2D monolayer (**B**) conditions. (**I**–**IV**) Gene expression analysis in 3D cultures: (**I**) *DNMT3A*, (**II**) *DNMT3B*, (**III**) *VIM*, and (**IV**) *E-cadherin*. (**V**–**VIII**) Gene expression analysis in 2D cultures: (**V**) *DNMT3A*, (**VI**) *DNMT3B*, (**VII**) *VIM*, and (**VIII**) *E-cadherin*. (* *p* < 0.05, ** *p* < 0.01).

**Figure 8:**
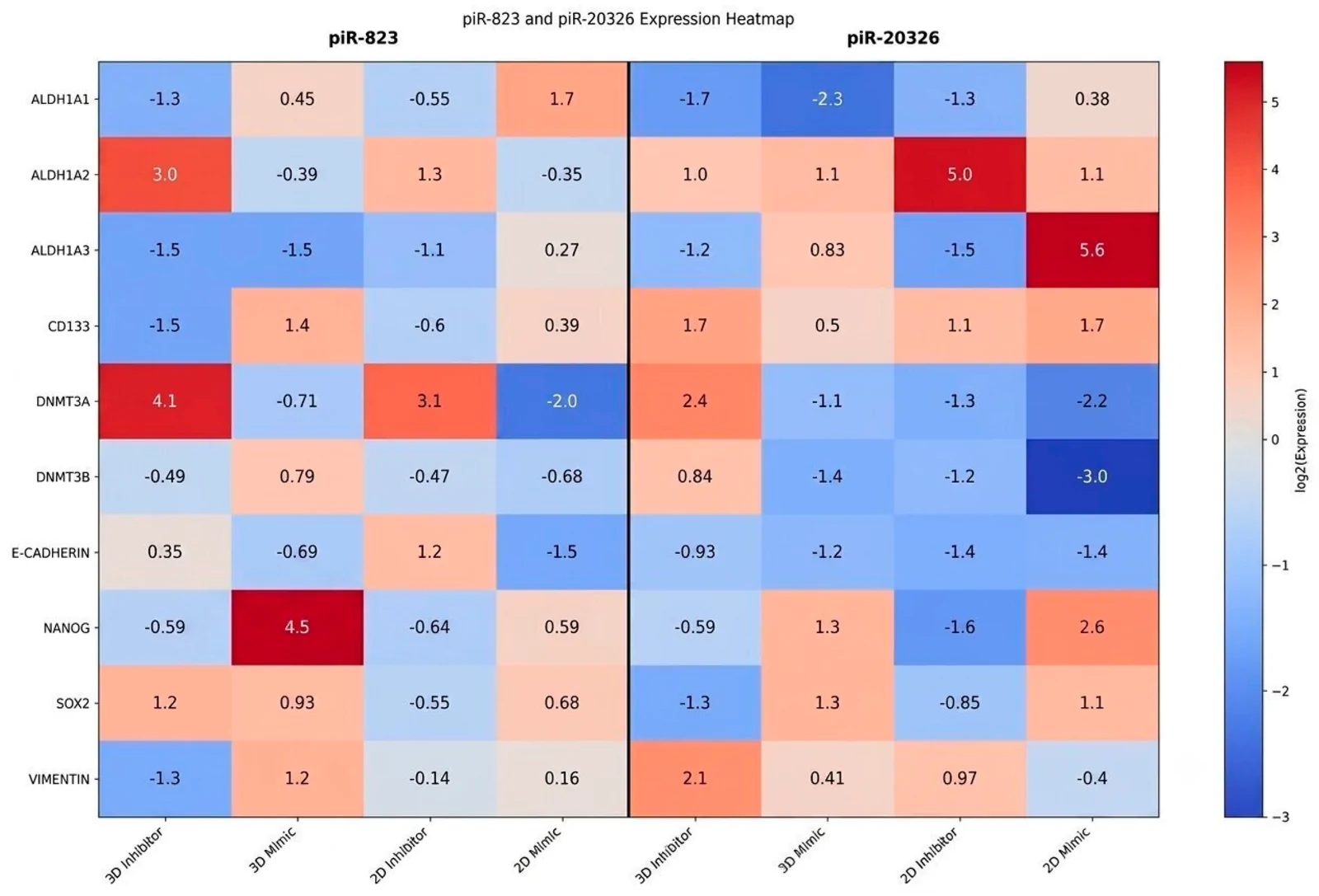
Heatmap visualization of stemness-related, EMT, and epigenetic marker expression following piR-823 and piR-20326 modulation in 2D and 3D ovarian cancer cell cultures.

**Figure 9:**
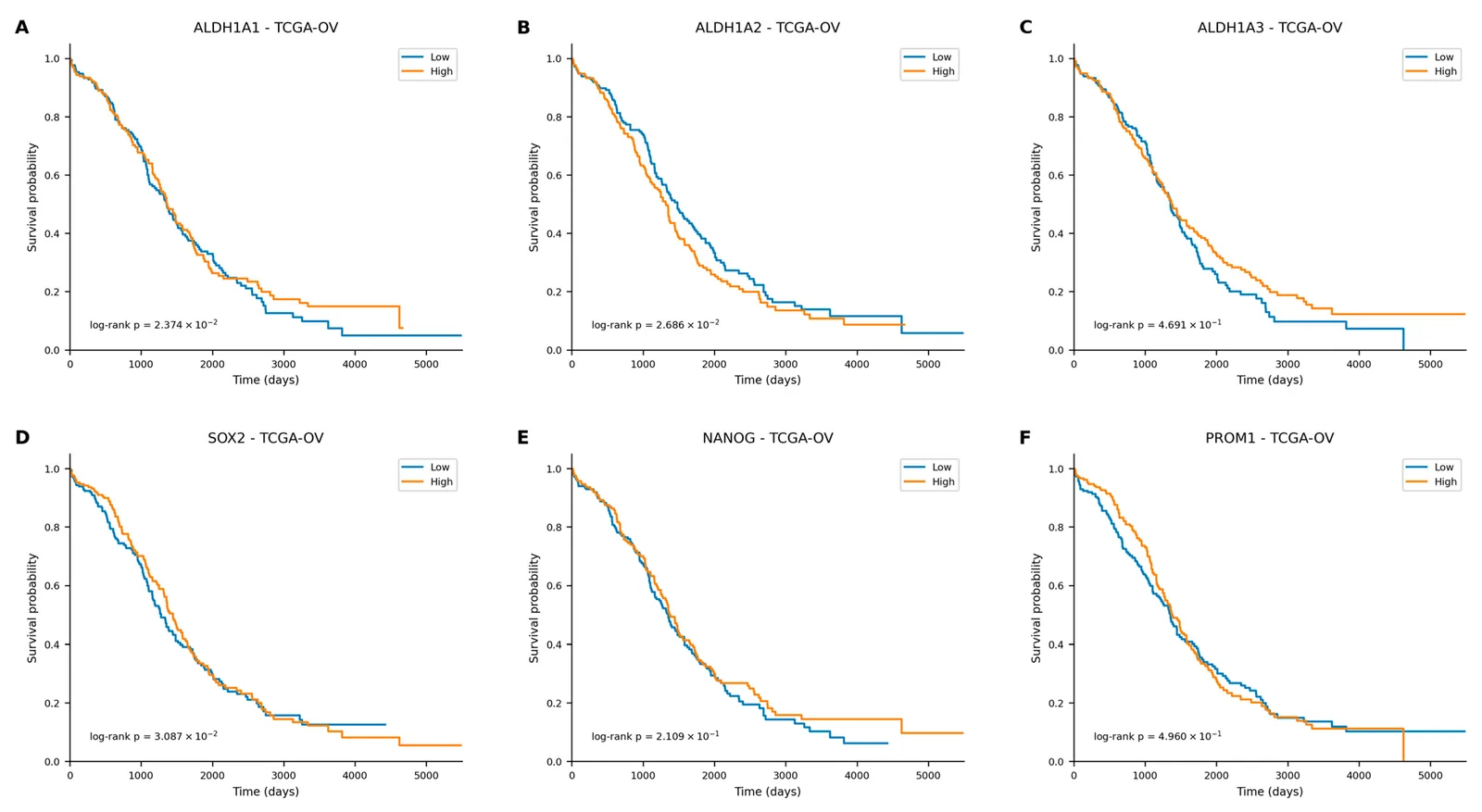
Kaplan–Meier survival analysis of stemness-associated genes in the TCGA-OV cohort. (**A**) Overall survival according to *ALDH1A1* expression; (**B**) overall survival according to *ALDH1A2* expression; (**C**) overall survival according to *ALDH1A3* expression; (**D**) overall survival according to *SOX2* expression; (**E**) overall survival according to *NANOG* expression; (**F**) overall survival according to *PROM1* expression. Patients were stratified into low- and high-expression groups. *p* values were determined using the log-rank test.

**Figure 10:**
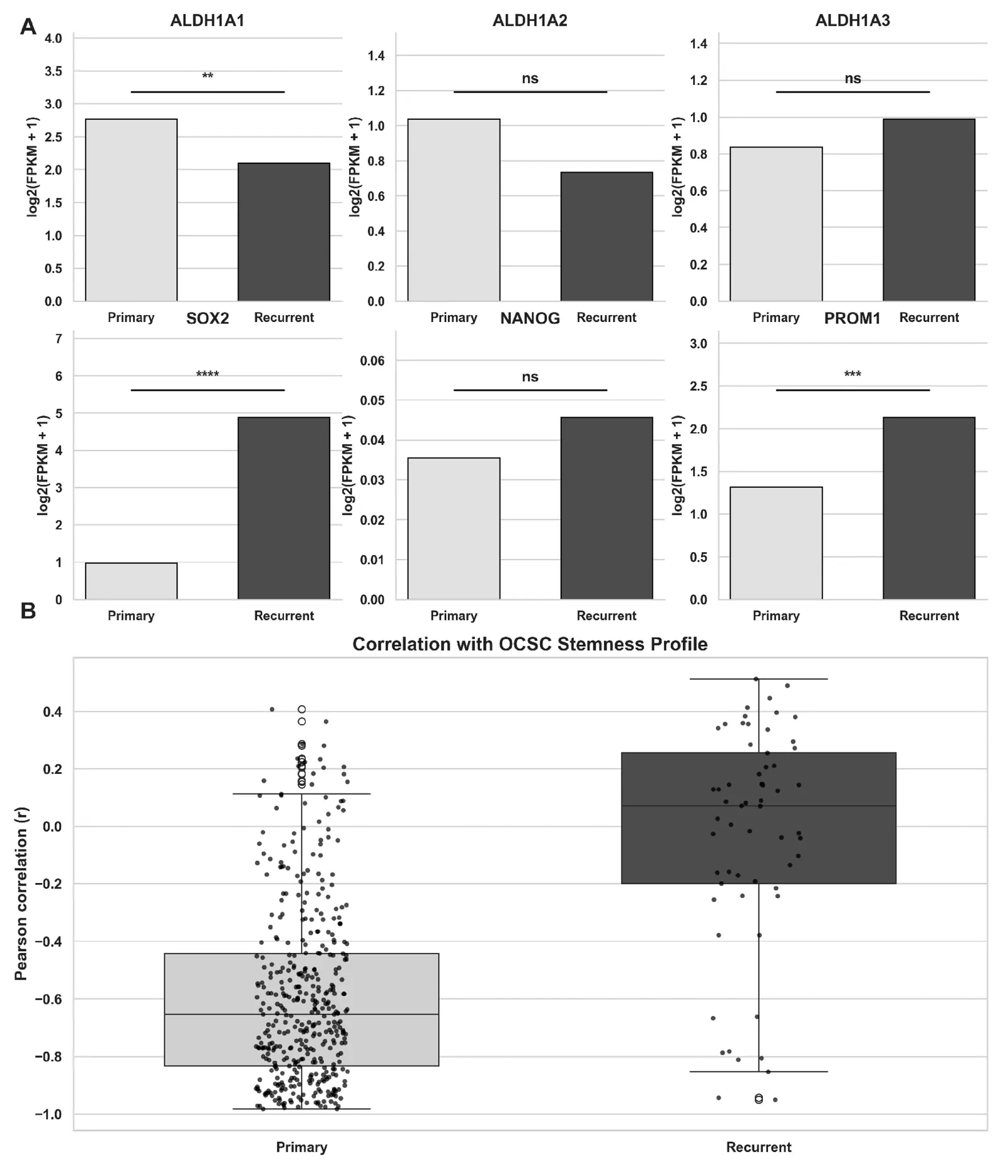
Expression and correlation analysis of stemness-related genes in primary and recurrent ovarian cancer. (**A**) Expression levels of ALDH1A1, ALDH1A2, ALDH1A3, SOX2, NANOG, and PROM1 in primary ovarian cancer (POC) and recurrent ovarian cancer (ROC). (**B**) Pearson correlation distribution of stemness-related gene expression in POC and ROC samples. Each dot represents the Pearson correlation coefficient between an individual stemness-related gene and the OCSC stemness profile for a given sample; open circles denote outlier values. (** *p* < 0.01, *** *p* < 0.001, **** *p* < 0.0001).

**Table 1:** The most differentially changed piRNAs and their relation to CSC.

piRNA	Seq (5′ to 3′)	PiRBase ID	Regulation in OC	FC	Relation with CSC (In The Literature)	CSC Related Gene Target (In Datasets)	Cancer Type	Reference
hsa_piRNA_30930	AGCGTTGGTGGTATAGTGGTGAGCATA	piR-31141	Down	0.0001383924884	-	No	BC, LC, M, NB, OC, RC, GC, HC, CC, PaC, PC, HNC, TC	[2]
hsa_piRNA_30929	AGCGTTGGTGGTATAGTGGTGAGCATAGC	piR-31142	Down	0.0001647858205	-	No	BC, LC, M, NB, OC, RC, GC, HC, CC, PaC, PC, HNC, CeC	[2]
hsa_piRNA_30928 (piR-823)	AGCGTTGGTGGTATAGTGGTGAGCATAGCTGC	piR-31143	Down	0.000265861085	Yes (piR-823 drives CSC traits via epigenetic regulation of Wnt/stemness pathways)	-	BC, LC, M, NB, OC, RC, GC, HC, CC, PaC, PC, HNC, CeC	[2,17]
hsa_piRNA_26089 (piR-20326)	GGCATTGGTGGTTCAGTGGTAGAATTCTCGC	piR-35982	Down	0.000320384		FLT1	BC, LC, M, NB, OC, RC, GC, HC, CC, PaC, PC, HNC, CeC	[2]
hsa_piRNA_27780	GACAATGAATACGTGTCTGGAACTCTGAGG	piR-34291	Down	0.003483464928	-	No	BC, LC, M, NB, OC, RC, GC, HC, CC, PaC, PC, HNC, TC	[2]
hsa_piRNA_49294	ATCTGTGATGTGAACCACAACCTGAGT	piRNA-L-52	Up	68.6347094694895	-	No	BC, LC, M, NB, OC, RC, GC, HC, CC, PaC, PC, HNC, CeC	[2]
hsa_piRNA_39053	AAACATATAACTGAACTCCTTACACC	-	Up	43.2103261807292	-	No	BC, LC, M, NB, OC, RC, GC, HC, CC, PaC, PC, HNC, CeC	[2]
hsa_piRNA_48781	AGGCTGTGATGGACCTGGCTGAGCCT	piRNA-L-489	Up	35.4294457670663	-	No	BC, LC, M, NB, OC, RC, GC, HC, CC, PaC, PC, HNC, CeC	[2]
hsa_piRNA_49127	ATAAATGATGATCAGCAGAATCTGAGT	piRNA-L-309	Up	33.7547751489292	-	No	BC, LC, M, NB, OC, RC, GC, HC, CC, PaC, PC, HNC, CeC	[2]
hsa_piRNA_49265	ATCTCTGATGACGCCTGTTCCTGAGA	hsa-piR-33103	Up	32.6296159772983	-	No	BC, LC, M, NB, OC, RC, GC, HC, CC, PaC, PC, HNC, CeC	[2]

## Data Availability

The RNA-seq expression and clinical data analyzed in this study were obtained from The Cancer Genome Atlas (TCGA-OV).

## Supplementary Materials

The following supporting information can be downloaded at https://www.mdpi.com/article/10.3390/ijms27198689/s1.
