# Supplementary material for: piR-823 and piR-20326 Differentially Regulate Stemness Programs in Ovarian Cancer Stem-like Cells
Source: Int J Mol Sci. 2026 Sep 29;27(19):8689. doi: 10.3390/ijms27198689 (PMC13665198; doi:10.3390/ijms27198689)
Supplement: Supplementary file 1 [file ijms-27-08689-s001.zip › ijms-4542769-supplementary file-final.pdf]

Supplementary material

Table S1. NCBI Accession numbers and sequences of selected piRNAs. siRNA sequences are designed to enhance piRNA silencing and expression.

piRNA	NCBI Accession No	PiRNA Seq (5′ to 3′)	Inhibitor siRNA Seq (5′ to 3′)	Mimic siRNA Seq (5′ to 3′)	Control Inhibitor siRNA Seq (5′ to 3′)	Control Mimic siRNA Seq (5′ to 3′)
piR-823	DQ571031	AGCGTT-GGTGG-TATAGTGGTGAG-CATAGCTGC	GCAGCUAUGCUCACCAC-UAUACCAC-CAACGCU	AGCGUUGGUG-GUAUAGUGGUG-AGCAUAGCUGC, AGCUAUGCUCAC-CACUAUACCAC-CAACGCU uu	GTCGTATCCAG-TGCGTGTCGTGGAG-TCGGCAATTGCAC-TGGATACGACTT-GTACTA	GTCGTATCCAG-TGCGTGTCGTG-GAGTCGGCAATT-GCACTGGATAC-GACAATTCTCCGA
piR-020326	DQ597916	GGCATT-GGTGGTTCAGTGG-TAGAATTCTCGC	GCGAGAAU-UCUAC-CACUGAAC-CAC-CAAUGCC	GGCAUUGGUG-GUUCAGUGGUA-GAAUUCUCGC, GAGAAUUCUAC-CACUGAACCAC-CAAUGCC uu	GTCGTATCCAG-TGCGTGTCGTGGAG-TCGGCAATTGCAC-TGGATACGACTT-GTACTA	GTCGTATCCAG-TGCGTGTCGTG-GAGTCGGCAATT-GCACTGGATAC-GACAATTCTCCGA

Table S2. The designed primer sequences of selected piRNAs.

Selected piRNA	Primers	Sequence of primers (5′ to 3′)
piR-823	Stem Loop	GTCGTATCCAGTGCGTGTCGTGGAGTCGGCAATTGCACTGGATACGACGCAGCTAT
	Forward	AACAGAAGCGTTGGTGGTATAGTGG
	Reverse	CAGTGCGTGTCGTGGAGT
piR-020326	Stem Loop	GTCGTATCCAGTGCGTGTCGTGGAGTCGGCAATTGCACTGGATACGACGCGAGAAT
	Forward	AACACGTGGGCATTGGTGGTT
	Reverse	CAGTGCGTGTCGTGGAGT
U6	Forward	CTCGCTTCGGCAGCACA
	Reverse	AACGCTTCACGAATTTGCGT
Negative control inhibitor siRNA	Forward	CAGTACTTTTGTGTAGTACAA
	Reverse	CAGTGCGTGTCGTGGAGT
Negative control mimic siRNA	Forward	TTCTCCGAACGTGTCACGTTTA
	Reverse	CAGTGCGTGTCGTGGAGT

Table S3. Designed primer sequences of selected stemness genes to demonstrate cancer stem cell characteristics.

Gene	Primers	Primer Sequence (5′-3′)
SOX2	SOX2_FP	GCCGAGTGGAAACTTTTGTCG
	SOX2_RP	GGCAGCGTGTACTTATCCTTCT
NANOG	NANOG_FP	TTTGTGGGCCTGAAGAAAACT
	NANOG_RP	AGGGCTGTCCTGAATAAGCAG
GAPDH	GAPDH_FP	CTGGGCTACACTGAGCACC
	GAPDH_RP	AAGTGGTCGTTGAGGGCAATG
CD133 (PROM1)	CD133_FP	GCTCCCTGTTGCTGCTG
	CD133_RP	TGGATTCATATGCCTTCTGT
ALDH1A1	ALDH1A1_FP	TGTTAGCTGATGGCCGACTTG
	ALDH1A1_RP	TTCTTAGCCCGCTCAACACT
ALDH1A2	ALDH1A2_FP	CCATTGGAGTGTGTGGACAG
	ALDH1A2_RP	GATGAGGGCTCCCATGTAGA
ALDH1A3	ALDH1A3_FP	TCTCGACAAAGCCCTGAAGT
	ALDH1A3_RP	GTCCGATGTTTGAGGAAGGA
DNMT3A	DNMT3A_FP	AGTACGACGACGACGGCTA
	DNMT3A_RP	CACACTCCACGCAAAAGCAC
DNMT3B	DNMT3B_FP	ACCTCGTGTGGGGAAAGATCA
	DNMT3B_RP	CCATCGCCAAACCACTGGA
E-cadherin	CDH1_FP	CCTTAGAGGTGGGTGACTAC
	CDH1_RP	CAAGAATCCCCAGAATGGCAG
Vimentin	VIM_FP	CGAAACTTCTCAGCATCACG
	VIM_RP	GCAGAAAGGCACTTGAAAGC

Table S4. The complete raw and BH-FDR-adjusted p-values of stemness-related genes, epigenetic markers, and EMT markers after piR-823 modulation.

Gene panel	Culture	Comparison	Gene	Raw p-value	BH-FDR adjusted p-value	Raw significance	FDR significance
Stemness	3D	Inhibitor vs Control	ALDH1A1	0.02	0.04	*	*
Stemness	3D	Inhibitor vs Control	ALDH1A2	0.004	0.012	**	*
Stemness	3D	Inhibitor vs Control	ALDH1A3	0.3	0.3	ns	ns
Stemness	3D	Inhibitor vs Control	CD133	0.004	0.012	**	*
Stemness	3D	Inhibitor vs Control	NANOG	0.3	0.3	ns	ns
Stemness	3D	Inhibitor vs Control	SOX2	0.12847	0.192705	ns	ns
Stemness	3D	Mimic vs Control	ALDH1A1	0.02	0.024	*	*
Stemness	3D	Mimic vs Control	ALDH1A2	0.1064	0.1064	ns	ns
Stemness	3D	Mimic vs Control	ALDH1A3	0.01	0.015	*	*
Stemness	3D	Mimic vs Control	CD133	0.002	0.006	**	**
Stemness	3D	Mimic vs Control	NANOG	0.0008	0.0048	***	**
Stemness	3D	Mimic vs Control	SOX2	0.005	0.01	**	*
Stemness	2D	Inhibitor vs Control	ALDH1A1	0.0004	0.0008	***	***
Stemness	2D	Inhibitor vs Control	ALDH1A2	0.01	0.012	*	*
Stemness	2D	Inhibitor vs Control	ALDH1A3	0.19246	0.19246	ns	ns
Stemness	2D	Inhibitor vs Control	CD133	0.0004	0.0008	***	***
Stemness	2D	Inhibitor vs Control	NANOG	0.0004	0.0008	***	***
Stemness	2D	Inhibitor vs Control	SOX2	0.01	0.012	*	*
Stemness	2D	Mimic vs Control	ALDH1A1	0.01	0.03	*	*
Stemness	2D	Mimic vs Control	ALDH1A2	0.01	0.03	*	*
Stemness	2D	Mimic vs Control	ALDH1A3	0.09955	0.11946	ns	ns
Stemness	2D	Mimic vs Control	CD133	0.07037	0.11946	ns	ns
Stemness	2D	Mimic vs Control	NANOG	0.52	0.52	ns	ns
Stemness	2D	Mimic vs Control	SOX2	0.09097	0.11946	ns	ns
Epigenetic_EMT	3D	Inhibitor vs Control	DNMT3A	0.007	0.014	**	*
Epigenetic_EMT	3D	Inhibitor vs Control	DNMT3B	0.03	0.03	*	*

Epigenetic_EMT	3D	Inhibitor vs Control	VIM	0.005	0.014	**	*
Epigenetic_EMT	3D	Inhibitor vs Control	E-cadherin	0.02	0.026666667	*	*
Epigenetic_EMT	3D	Mimic vs Control	DNMT3A	0.009	0.018	**	*
Epigenetic_EMT	3D	Mimic vs Control	DNMT3B	0.04	0.04	*	*
Epigenetic_EMT	3D	Mimic vs Control	VIM	0.005	0.018	**	*
Epigenetic_EMT	3D	Mimic vs Control	E-cadherin	0.02	0.026666667	*	*
Epigenetic_EMT	2D	Inhibitor vs Control	DNMT3A	0.001	0.004	**	**
Epigenetic_EMT	2D	Inhibitor vs Control	DNMT3B	0.97	0.97	ns	ns
Epigenetic_EMT	2D	Inhibitor vs Control	VIM	0.8	0.97	ns	ns
Epigenetic_EMT	2D	Inhibitor vs Control	E-cadherin	0.75	0.97	ns	ns
Epigenetic_EMT	2D	Mimic vs Control	DNMT3A	0.001	0.004	**	**
Epigenetic_EMT	2D	Mimic vs Control	DNMT3B	0.57	0.88	ns	ns
Epigenetic_EMT	2D	Mimic vs Control	VIM	0.88	0.88	ns	ns
Epigenetic_EMT	2D	Mimic vs Control	E-cadherin	0.76	0.88	ns	ns

Table S5. The complete raw and BH-FDR-adjusted p-values of stemness-related genes, epigenetic markers, and EMT markers after piR-20326 modulation.

Panel	Culture	Comparison	Gene	Raw_p	BH_FDR_adjusted_p	Raw_symbol	FDR_symbol
Stemness	3D	Inhibitor vs Control	ALDH1A1	0.006	0.018	**	*
Stemness	3D	Inhibitor vs Control	ALDH1A2	0.05528	0.05528	ns	ns
Stemness	3D	Inhibitor vs Control	ALDH1A3	0.02	0.03	*	*
Stemness	3D	Inhibitor vs Control	CD133	0.03	0.036	*	*
Stemness	3D	Inhibitor vs Control	NANOG	0.005	0.018	**	*
Stemness	3D	Inhibitor vs Control	SOX2	0.01	0.02	*	*
Stemness	3D	Mimic vs Control	ALDH1A1	0.015	0.0225	*	*
Stemness	3D	Mimic vs Control	ALDH1A2	0.001	0.003	**	**
Stemness	3D	Mimic vs Control	ALDH1A3	0.01	0.02	*	*
Stemness	3D	Mimic vs Control	CD133	0.05581	0.05581	ns	ns
Stemness	3D	Mimic vs Control	NANOG	0.04	0.048	*	*
Stemness	3D	Mimic vs Control	SOX2	0.001	0.003	**	**
Stemness	2D	Inhibitor vs Control	ALDH1A1	0.21197	0.21197	ns	ns
Stemness	2D	Inhibitor vs Control	ALDH1A2	0.0005	0.0015	***	**
Stemness	2D	Inhibitor vs Control	ALDH1A3	0.0005	0.0015	***	**
Stemness	2D	Inhibitor vs Control	CD133	0.08627	0.15	ns	ns
Stemness	2D	Inhibitor vs Control	NANOG	0.1	0.15	ns	ns
Stemness	2D	Inhibitor vs Control	SOX2	0.2	0.21197	ns	ns
Stemness	2D	Mimic vs Control	ALDH1A1	0.1	0.1575	ns	ns
Stemness	2D	Mimic vs Control	ALDH1A2	0.3	0.3	ns	ns
Stemness	2D	Mimic vs Control	ALDH1A3	0.0001	0.0006	***	***
Stemness	2D	Mimic vs Control	CD133	0.15	0.18	ns	ns
Stemness	2D	Mimic vs Control	NANOG	0.105	0.1575	ns	ns
Stemness	2D	Mimic vs Control	SOX2	0.0033	0.0099	**	**
Epigenetic_EMT	3D	Inhibitor vs Control	DNMT3A	0.1	0.177333	ns	ns
Epigenetic_EMT	3D	Inhibitor vs Control	DNMT3B	0.0597	0.177333	ns	ns
Epigenetic_EMT	3D	Inhibitor vs Control	VIM	0.201	0.201	ns	ns
Epigenetic_EMT	3D	Inhibitor vs Control	E-cadherin	0.133	0.177333	ns	ns
Epigenetic_EMT	3D	Mimic vs Control	DNMT3A	0.51	0.51	ns	ns
Epigenetic_EMT	3D	Mimic vs Control	DNMT3B	0.083	0.332	ns	ns
Epigenetic_EMT	3D	Mimic vs Control	VIM	0.25	0.333333	ns	ns
Epigenetic_EMT	3D	Mimic vs Control	E-cadherin	0.194	0.333333	ns	ns
Epigenetic_EMT	2D	Inhibitor vs Control	DNMT3A	0.189	0.252	ns	ns
Epigenetic_EMT	2D	Inhibitor vs Control	DNMT3B	0.491	0.491	ns	ns
Epigenetic_EMT	2D	Inhibitor vs Control	VIM	0.098	0.196	ns	ns
Epigenetic_EMT	2D	Inhibitor vs Control	E-cadherin	0.066	0.196	ns	ns
Epigenetic_EMT	2D	Mimic vs Control	DNMT3A	0.288	0.576	ns	ns
Epigenetic_EMT	2D	Mimic vs Control	DNMT3B	0.58	0.6	ns	ns
Epigenetic_EMT	2D	Mimic vs Control	VIM	0.287	0.576	ns	ns
Epigenetic_EMT	2D	Mimic vs Control	E-cadherin	0.6	0.6	ns	ns

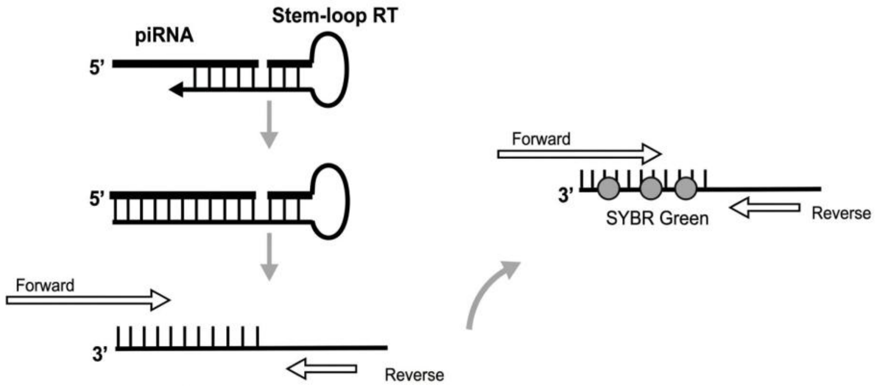

Figure S1. Stem-loop primer strategy for piRNA amplification.

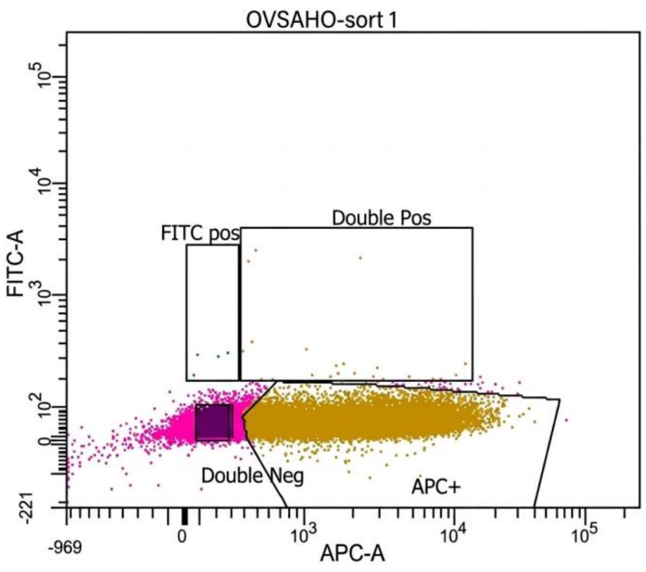

Figure S2. Gating strategy for cells separated from OVSAHO by FACS.

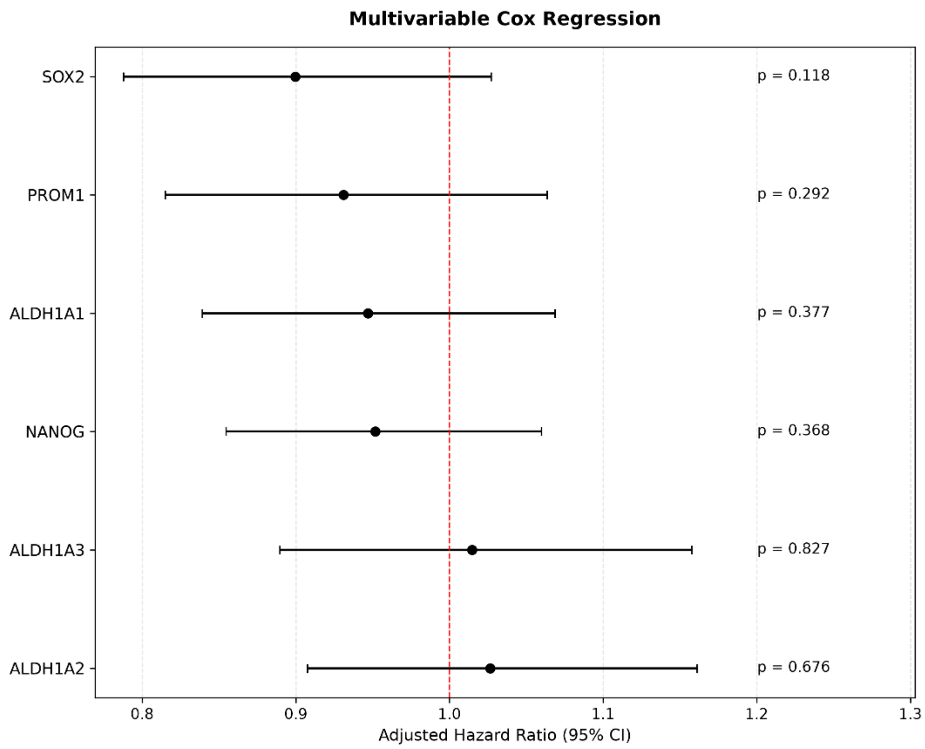

Figure S3. Multivariable Cox regression analysis of stemness-associated genes in ovarian cancer. Adjusted hazard ratios (HRs) and 95% confidence intervals (CIs) are shown.

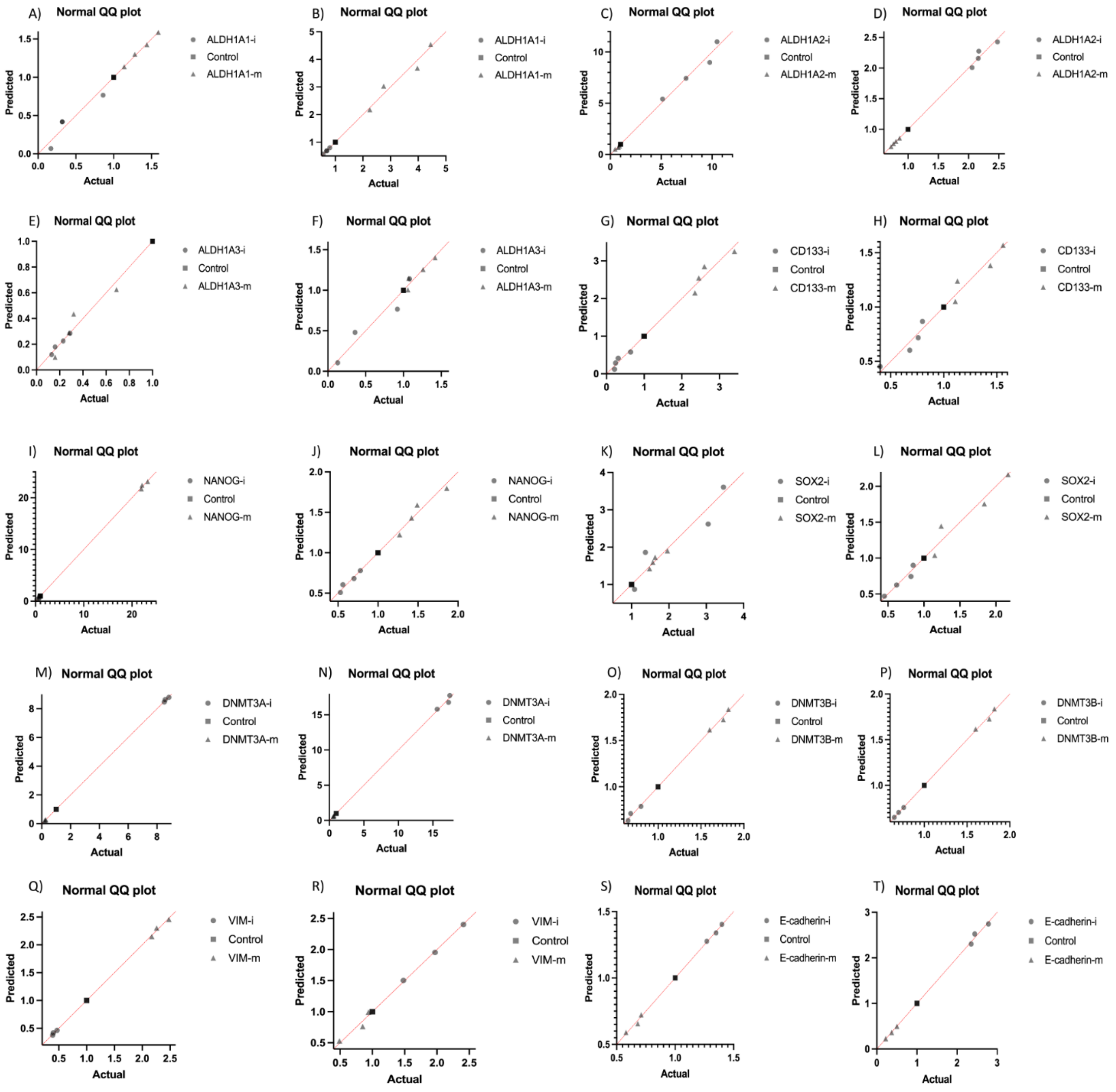

Figure S4. Normal Q–Q plots for assessing data distribution in piR-823 experiments. Normal Q–Q plots generated in GraphPad Prism are shown for relative gene-expression data following piR-823 inhibition (i) and overexpression (m) compared with the corresponding control in 3D spheroid and 2D monolayer cultures. Panels represent the following genes and culture conditions: (A) *ALDH1A1*, 3D spheroid; (B) *ALDH1A1*, 2D monolayer; (C) *ALDH1A2*, 3D spheroid; (D) *ALDH1A2*, 2D monolayer; (E) *ALDH1A3*, 3D spheroid; (F) *ALDH1A3*, 2D monolayer; (G) *CD133* (*PROM1*), 3D spheroid; (H) *CD133* (*PROM1*), 2D monolayer; (I) *NANOG*, 3D spheroid; (J) *NANOG*, 2D monolayer; (K) *SOX2*, 3D spheroid; (L) *SOX2*, 2D monolayer; (M) *DNMT3A*, 3D spheroid; (N) *DNMT3A*, 2D monolayer; (O) *DNMT3B*, 3D spheroid; (P) *DNMT3B*, 2D monolayer; (Q) Vimentin (VIM), 3D spheroid; (R) Vimentin (VIM), 2D monolayer; (S) E-cadherin (*CDH1*), 3D spheroid; and (T) E-cadherin (*CDH1*), 2D monolayer. Circles represent inhibitor-transfected samples (i), squares represent control samples, and triangles represent mimic-transfected samples (m). The reference line represents the expected relationship under a normal distribution.

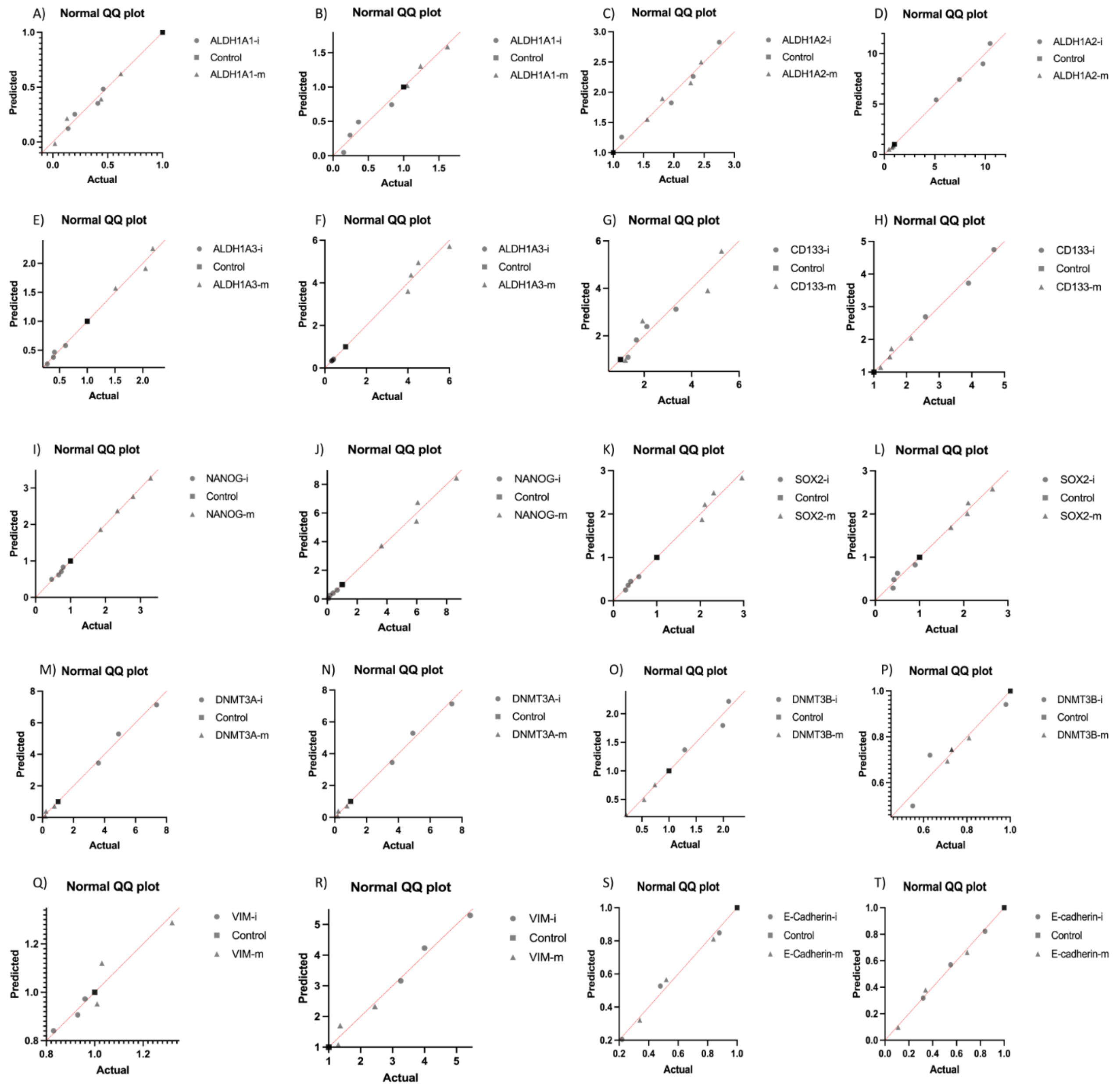

Figure S5. Normal Q–Q plots for the assessment of data distribution in piR-20326 experiments. Normal Q–Q plots generated in GraphPad Prism are shown for relative gene-expression data following piR-20326 inhibition (i) and overexpression (m) compared with the corresponding control in 3D spheroid and 2D monolayer cultures. Panels represent the following genes and culture conditions: (A) *ALDH1A1*, 3D spheroid; (B) *ALDH1A1*, 2D monolayer; (C) *ALDH1A2*, 3D spheroid; (D) *ALDH1A2*, 2D monolayer; (E) *ALDH1A3*, 3D spheroid; (F) ALDH1A3, 2D monolayer; (G) *CD133* (PROM1), 3D spheroid; (H) *CD133* (*PROM1*), 2D monolayer; (I) *NANOG*, 3D spheroid; (J) *NANOG*, 2D monolayer; (K) *SOX2*, 3D spheroid; (L) *SOX2*, 2D monolayer; (M) *DNMT3A*, 3D spheroid; (N) *DNMT3A*, 2D monolayer; (O) *DNMT3B*, 3D spheroid; (P) *DNMT3B*, 2D monolayer; (Q) *Vimentin* (VIM), 3D spheroid; (R) *Vimentin* (VIM), 2D monolayer; (S) *E-cadherin* (CDH1), 3D spheroid; and (T) *E-cadherin* (CDH1), 2D monolayer. Circles represent inhibitor-transfected samples (i), squares represent control samples, and triangles represent mimic-transfected samples (m). The reference line represents the expected relationship under a normal distribution.
